# Landscape and evolution of tissue-specific alternative polyadenylation across *Drosophila* species

**DOI:** 10.1186/s13059-017-1358-0

**Published:** 2017-11-30

**Authors:** Piero Sanfilippo, Jiayu Wen, Eric C. Lai

**Affiliations:** 1 Department of Developmental Biology, Sloan-Kettering Institute, New York, New York 10065 USA; 2 Louis V. Gerstner, Jr. Graduate School of Biomedical Sciences, Memorial Sloan Kettering Cancer Center, New York, New York 10065, USA; 3 Present address: Biochemistry and Biomedical Sciences, Research School of Biology, ANU College of Science, The Australian National University, Canberra, ACT 2601 Australia

## Abstract

**Background:**

*Drosophila melanogaster* has one of best-described transcriptomes of any multicellular organism. Nevertheless, the paucity of 3′-sequencing data in this species precludes comprehensive assessment of alternative polyadenylation (APA), which is subject to broad tissue-specific control.

**Results:**

Here, we generate deep 3′-sequencing data from 23 developmental stages, tissues, and cell lines of *D. melanogaster*, yielding a comprehensive atlas of ~ 62,000 polyadenylated ends. These data broadly extend the annotated transcriptome, identify ~ 40,000 novel 3′ termini, and reveal that two-thirds of *Drosophila* genes are subject to APA. Furthermore, we dramatically expand the numbers of genes known to be subject to tissue-specific APA, such as 3′ untranslated region (UTR) lengthening in head and 3′ UTR shortening in testis, and characterize new tissue and developmental 3′ UTR patterns. Our thorough 3′ UTR annotations permit reassessment of post-transcriptional regulatory networks, via conserved miRNA and RNA binding protein sites. To evaluate the evolutionary conservation and divergence of APA patterns, we generate developmental and tissue-specific 3′-seq libraries from *Drosophila yakuba* and *Drosophila virilis*. We document broadly analogous tissue-specific APA trends in these species, but also observe significant alterations in 3′ end usage across orthologs. We exploit the population of functionally evolving poly(A) sites to gain clear evidence that evolutionary divergence in core polyadenylation signal (PAS) and downstream sequence element (DSE) motifs drive broad alterations in 3′ UTR isoform expression across the *Drosophila* phylogeny.

**Conclusions:**

These data provide a critical resource for the *Drosophila* community and offer many insights into the complex control of alternative tissue-specific 3′ UTR formation and its consequences for post-transcriptional regulatory networks.

**Electronic supplementary material:**

The online version of this article (doi:10.1186/s13059-017-1358-0) contains supplementary material, which is available to authorized users.

## Background

For the past century, *Drosophila melanogaster* has served as one of the premier metazoan model organism systems, and its practitioners have been at the forefront for advancing genetic tools and genomic knowledge. Accordingly, this species has one of the best-understood transcriptomes of any animal species. During the course of large-scale studies such as the Berkeley *Drosophila* Genome Project, the modENCODE project, and allied community efforts, various technologies were employed to catalog *Drosophila* gene expression with progressively deeper profiling and more exacting and precise resolution. These include cDNA sequencing [1], gene and whole genome microarrays [2, 3], mRNA-sequencing [4–6], total RNA-sequencing [7, 8], and small RNA sequencing [9, 10], which have now been applied to hundreds of tissue and cell samples, mutant conditions, and/or environmental perturbations. The numbers of non-coding transcripts, especially of small regulatory RNAs and testis lncRNAs, have changed dramatically over the years. The numbers of protein-coding gene loci have also increased to some extent, but the diversity in the conventional transcriptome has mostly come from the appreciation of isoform diversity. This can come from processes such as differential promoter usage [11–13], alternative forward splicing [4, 14], back splicing [7], and alternative polyadenylation (APA) [6]. Moreover, these processes can act combinatorially to diversify transcript output from a given locus.

While RNA-sequencing (RNA-seq) provides tremendous depth and insight into the transcriptome, due to the nature of underlying strategies for library preparation, general RNA-seq methods are deficient in resolving transcript termini [15]. To some extent, this can be remedied by custom algorithms for interpreting RNA-seq data [16], but specialized methods for sequencing 5′ and 3′ transcript termini are ultimately needed to gain requisite information. In *Drosophila*, a substantial amount of CAGE-sequencing across developmental and tissue samples has been performed to annotate transcription start sites [11–13]. On the other hand, despite the appreciation of pervasive alternative 3′ end usage in *Drosophila*, including broad patterns of tissue-specific APA [6, 17], a broad compendium of deep 3′-sequencing (3′-seq) data across tissues is still lacking in this species. The application of 3′-seq approaches in diverse other organisms has been invaluable not only for defining transcript termini precisely, but for revealing unexpected APA trends [18]. However, available 3′-seq data for *Drosophila* have only begun to emerge recently, and these sample limited tissue settings [19, 20].

3′ Untranslated regions (3′ UTRs) are predominant mediators of post-transcriptional regulation, and the phenomenon of APA can render such regulation conditional upon developmental stage, tissue type, physiological status, environmental state, and so forth [18, 21]. For example, there are several locations of broad, tissue-specific deployment of tandem polyadenylation sites within 3′ UTRs. These include the usage of many distal sites in the nervous system of invertebrates and vertebrates, and the usage of many proximal sites in gonads, including in *Drosophila* and mammalian testis, and in zebrafish ovary [6, 17, 22, 23]. Given that 3′ UTRs are the main hub for post-transcriptional regulation, APA has a broad impact on the regulation and function of the affected transcripts [24]. Since the expression of different 3′ UTR isoforms is influenced by cellular [25], tissue [26], and developmental state [27] and dysregulated in disease [28], APA collectively has broad consequences on gene regulation and biology.

In this study, we complete a missing aspect of the *D. melanogaster* transcriptome resource with a deep and broad compendium of 3′-seq data across a timecourse of embryogenesis, multiple adult tissues, and a panel of cell lines, yielding a catalog of ~ 62,000 confident termini of polyadenylated transcripts. Analysis of these tissues covers the majority of described APA diversity and permits discovery of a wealth of novel transcript isoforms, despite recent intense profiling of the *Drosophila* transcriptome [8]. We find evidence of APA for two-thirds of all genes and report novel tissue-specific expression of 3′ UTR isoforms for thousands of genes. We broadly extend the finding that head and testis exhibit characteristic APA patterns, and identify the ovary as a new setting for 3′ UTR shortening in *Drosophila*. Our 3′ UTR revisions serve as the basis of revising the catalogs of conserved miRNA and RNA binding protein sites, which can be subject to alternative regulation. As it is unknown to what extent Drosophilid patterns of tissue-specific APA are under constraint, we generated extensive developmental and tissue-specific 3′-seq datasets from close (*Drosophila yakuba*) and distant (*Drosophila virilis*) fruit fly relatives. Comparison of tissue-specific patterns of 3′ UTR expression between species shows conservation of expression of long 3′ UTR isoforms in head and suggests a faster rate of evolution for testis-specific events. Finally, we present a deep analysis of pA site conservation between species and provide broad empirical evidence that *cis*-element changes play determinant roles in the evolution of patterns of pA site usage and 3′ UTR isoform expression.

Overall, these comprehensive, multi-species 3′ UTR and regulatory site annotations provide an invaluable resource for the *Drosophila* community and yield diverse insights into the breadth and evolution of post-transcriptional regulatory networks across this key model organism clade.

## Results

### Atlas of polyadenylated termini across *D. melanogaster* development, tissues, and cell lines

To assess the landscape of 3′ UTR isoforms in *D. melanogaste*r, we initially generated parallel total RNA sequencing (RNA-seq) and 3′-sequencing (3′-seq) data from dissected female heads, ovaries, and testes, a compact set of tissues that efficiently samples both transcript [5] and 3′ UTR diversity [6]. 3′-Seq data identify sites of cleavage and polyadenylation (CPA) at nucleotide resolution via reads that exhibit untemplated stretches of adenosines (As) at their 3′ termini, thereby indicating polyadenylation at the cleavage site.

**Fig. 1:**
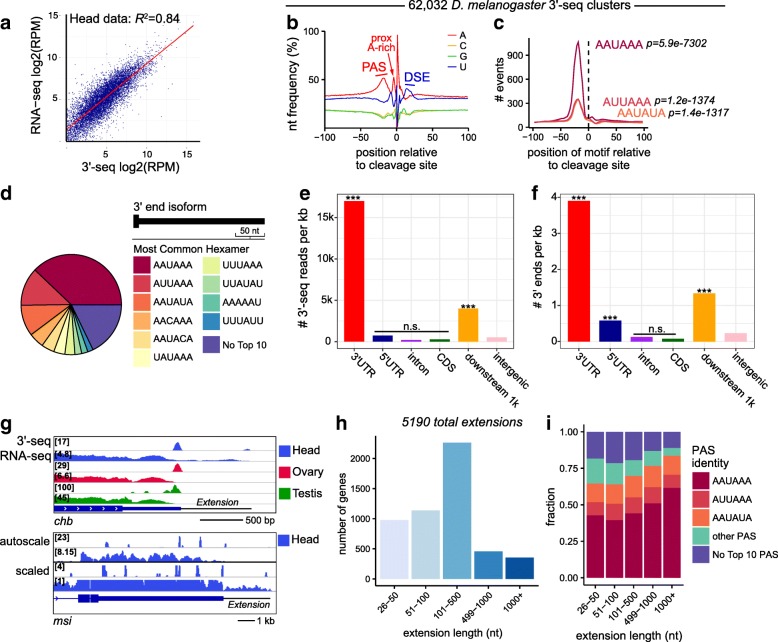
Properties of the *D. melanogaster* 3′-seq atlas. **a** 3′-Seq is a quantitative measure of gene expression. Correlation of gene expression measurements derived using either RNA-seq or 3′-seq of matched female head samples. Pearson correlation coefficient is reported. *Red line* represents linear regression fit. **b** 3′-Seq-derived 3′ ends are aligned around the predicted cleavage site and the nucleotide distribution around putative cleavage site is plotted (centered at 0). Percentages of each of four nucleotides in a window of 200 bp centered at the cleavage site are shown. Location of A-rich PAS and U-rich downstream sequence element are shown as well as insect-specific A-rich sequence ~ 5 nucleotides upstream of the cleavage site. **c** Positional enrichment of the top three hexamers found in a ±100-nucleotide window around cleavage sites. **d** De novo derivation of most represented hexamers in a 50-nucleotide window upstream of 3′-seq-derived 3′ end positions in terminal 3′ UTRs. **e, f** Distribution of 3′-seq reads (**e**) and derived 3′ end sites (**f**) onto existing genomic features in the *D. melanogaster* annotation (r6.12). Statistical significance test of each region against intergenic space was performed by determining total number of reads (**e**) or 3′ ends (**f**) in each region relative to their proportion of base frequencies, which was assessed by binomial tests. *** p < 0.0005; n.s. p > 0.05. **g** Examples of 3′ end extensions of current FlyBase r6.12 gene models. RNA-seq and 3′-seq IGV tracks are shown in autoscale. A head-specific extension is evident for *chb*. An additional set of scaled tracks is shown for *msi*, to highlight the low, but discrete, expression of its extension which terminates with a defined 3′-seq cluster. **h** Barplot summarizing the numbers of 3′ UTR extensions of FlyBase r6.12 gene models that were made on the basis of downstream 3′-seq clusters connected by RNA-seq evidence. **i** Quality of PAS motifs associated with novel 3′ UTR extensions to FlyBase r6.12 gene models. A higher frequency of AAUAAA is observed at the most distal extensions

To evaluate whether our 3′-seq data reported gene expression quantitatively, we compared gene expression values obtained from the two sequencing methodologies. Plotting the normalized numbers of total RNA-seq reads and 3′-seq reads mapped to individual gene models revealed strong linear concordance over 4 orders of magnitude (*R* ^2^ = 0.81–0.85) between the datasets for each tissue type (Fig. 1a; Additional file 1: Figure S1). With these confirmations in hand, we extended our analysis by generating additional 3′-seq data from whole adult flies and carcass, an embryonic timecourse (0–45′, 45′–90′, 90′–6 h, 6–12 h, 12–18 h, and 18–24 h), and a panel of ten cultured cell lines (Additional file 2: Table S1). We used principal components analysis (PCA) of the 3′-seq libraries and comparable RNA-seq datasets (Additional file 2: Table S1) to visualize both the overall concordance of these data types and the diversity of these libraries (Additional file 1: Figure S2).

Collectively, these 3′-seq libraries comprise ~ 200 million mapped reads from which we could call polyadenylation (pA) sites. Importantly, a strong majority (78%) of the reads mapped onto known 3′ UTRs (Additional file 1: Figure S3). This provides clear indication of the quality of these libraries as reflecting termini of messenger RNA. In aggregate, these samples allowed us to capture > 97% of all *Drosophila* genes annotated in *D. melanogaster* (r6.12), with 83% of all genes expressed at a minimum of 1 RPM in at least one library. We identified pA sites using reads bearing segments of untemplated poly(A). We filtered pA sites with downstream A-rich stretches of nucleotides to minimize potential sites of internal priming, and called dominant pA events when the events fell within 25 nucleotides of each other [29]. We report here pA sites that are supported by at least three distinct reads amongst all libraries. In total, we confidently identify 62,032 pA sites (Additional file 3: Table S2; Additional file 4), of which ~ 40,000 are novel with respect to FlyBase annotation (r6.12). Therefore, our 3′-seq datasets provide comprehensive data on the landscape of 3′ end formation across the *Drosophila* transcriptome.

The CPA machinery recognizes specific *cis*-regulatory motifs and sequence context to determine sites of 3′ end formation, most notably the core polyadenylation signal (PAS; AAUAAA and other rarer variants) usually located 10–40 nucleotides upstream of the cleavage site, and a U-rich downstream sequence element (DSE) in the ~ 20 nucleotides following the cleavage site [30]. We examined the sequence composition surrounding pA sites in our atlas, and observed strong enrichment of characteristic A-rich upstream PAS and U-rich DSE in the appropriate sequence ranges with respect to cleavage sites (Fig. 1b). Interestingly, we also observe a distinct A-rich sequence immediately upstream (< 5 nucleotides) of the cleavage site (Fig. 1b). This feature was previously noted from analysis of fly and mosquito 3′ ends derived from EST data [31–33], but has not subsequently been observed in any of the many other organisms analyzed by 3′-seq [30]. We performed de novo motif searches in a ± 100-nucleotide window surrounding the ~ 62,000 cleavage sites, and observed overwhelming enrichment of canonical PAS and variant signals peaking at the −20-nucleotide position (Fig. 1c). The distribution of most common motifs in the 50-nucleotide window upstream of ends mapping to 3′ UTRs is shown in Fig. 1d. Interestingly, the order of the top four motifs (AAUAAA > AUUAAA > AAUAUA > UAUAAA) is the same as earlier reported from *Drosophila* EST analysis [32, 33]. Although the dominance of AWUAAA hexamers was expected, the usage of AAUAUA as the functional PAS in ~ 10% of *Drosophila* 3′ termini was notable, since it was not observed as a substantial PAS variant in deep sequencing of 3′ ends from *Caenorhabditis elegans* [34] or human [35].

Taken together, the sequence and motif properties validate the legitimacy of our 3′ termini maps and highlight idiosyncrasies of insect 3′ termini. Overall, these data permit high quality, nucleotide resolution, quantitative identification of the 3′ ends of polyadenylated RNAs, and the breadth and depth of our atlas comprises the most comprehensive such catalog of pA sites in *D. melanogaster* to date.

### 3′-Seq atlas broadly extends *D. melanogaster* gene annotations and isoforms

We examined the properties of our 3′-seq atlas in further detail. Interestingly, amongst the population of apparent intergenic raw 3′-seq reads, a disproportionate fraction of them mapped on the sense strand downstream of existing gene models. For example, more raw 3′-seq reads mapped to the sense strand 1 kb downstream of FlyBase r6.12 gene models than to either strand of the remainder of intergenic space (Additional file 1: Figure S3) and this enrichment was enhanced when we consider the positional enrichment of our 3′ end calls (Additional file 1: Figure S3). This was even more striking when normalizing for the disparate size of these genomic compartments (Fig. 1e, f; *p* ≈ 0, binomial test). This reflects that the genomic region immediately downstream of protein-coding genes contains a smaller number of higher-expressed poly(A) sites whereas total intergenic space contains many more marginally expressed 3′-seq clusters. Although the *D. melanogaster* transcriptome has arguably been quite thoroughly annotated via the modENCODE project [5, 36], these observations suggested that the most distal 3′ ends of many protein-coding genes might still remain under-annotated.

In order to assign these putative 3′ ends to upstream transcription units, we leveraged RNA-seq as additional evidence to aid in attributing the ends. Briefly, we assembled gene models de novo using isoSCM [16], and required that the sequence between the end of the annotation and the 3′-seq cluster have continuous RNA-seq evidence with any gaps < 100 bp. For example, *chb* shows extensions of its model by ~ 850 nucleotides, as evidenced by both 3′-seq and RNA-seq signals in samples from heads (Fig. 1g).

Remarkably, we identify 5190 *D. melanogaster* r6.12 gene models whose 3′ termini can be extended by > 25 nucleotides, and nearly a thousand of these can be extended by > 500 nucleotides (Fig. 1h). In total, this adds ~ 1.6 Mb to the transcriptome of protein-coding genes. The majority of these novel extension events, especially the distant extensions, represent minor isoforms in terms of bulk transcript. For example, the novel long 3′ UTR extension of the gene *msi* represents < 1% of total *msi* transcript in head (Fig. 1g). Nevertheless, the fraction of canonical PAS (AAUAAA) increases in the most distant, novel pA sites (0.5–1 kb and 1 kb + extensions), compared to novel pA sites that yield shorter extensions (56 vs 43%, *p* = 1.2E-11, Fisher’s exact test) (Fig. 1i). A linear regression analysis of the fraction of AAUAAA showed an increase with extension length (*p* = 0.002, *R* ^2^ = 0.96), confirming the validity of these events. As explored later, functional relevance of at least a subset of these is indicated by the fact that they harbor well-conserved post-transcriptional regulatory sites. Thus, these lower expressed 3′ UTR isoforms deserve annotation (Additional file 5: Table S3), and they may potentially represent highly regulated and/or cell type restricted transcripts.

### Complexity of APA in *D. melanogaster* across cells and tissues

**Fig. 2:**
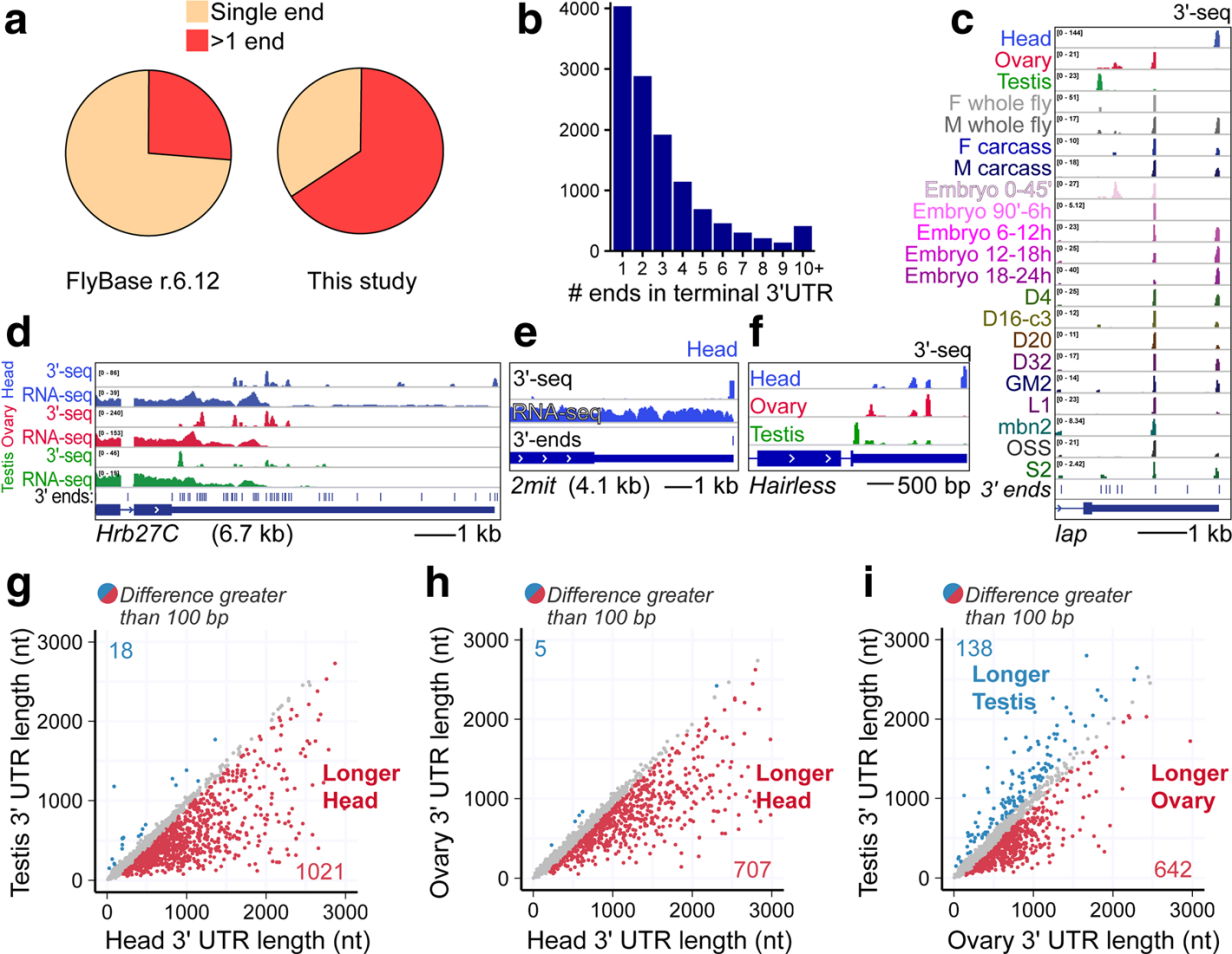
Tissue-specific alternative polyadenylation in *D. melanogaster*. **a** Fraction of genes with one or more annotated 3′ ends in the current FlyBase annotation (r6.12) compared to our revised 3′-seq-based atlas. **b** Summary of 3′ terminal isoform numbers annotated for genes in our 3′-seq-based atlas. **c–f** Examples of genes with diverse APA patterns. **c** Example of a gene with complex pattern of 3′ end isoform expression across adult tissues, embryonic timecourse, and cell lines. **d** Example of a gene with a long 3′ UTR that exhibits extreme 3′ end diversity. **e** Example of a gene with a long (4 kb) 3′ UTR that generates a single 3′ end in head. **f** Example of a gene with distinctive tissue-specific APA isoforms deployed in testis, ovary, and head. **g–i** Genome-wide patterns of tissue-specific APA revealed by pairwise analyses of weighted 3′ UTR length (denoted as 3′ UTR length for simplicity) comparison between tissues. Weighted 3′ UTR length is obtained taking the average of all 3′ UTR isoform lengths per gene weighted by the contribution of each isoform expressed. Genes are expressed at a minimum of 5 RPM in all samples. The genes for which weighted 3′ UTR length differs by 100 bp or more between samples are shown colored: *red*, longer weighted length in the sample on the *x-axis*; *blue*, longer weighted length in the sample on the *y-axis*. **g** Female head vs. testis. **h** Female head vs. ovary. **i** Ovary vs. testis

Including our novel 3′ end annotations that extend known gene models, we expand the number of genes that undergo APA from ~ 25% currently noted in FlyBase r6.12 to ~ 65% (Fig. 2a), a proportion similar to that observed in other eukaryotes [26]. Indeed, the complexity of 3′ end isoform expression in *Drosophila* is underscored by the fact that ~ 25% of genes express four or more 3′ end isoforms (Fig. 2b). We emphasize the utility of our atlas with examples such as *lap*, *Cul-1*, and *Xpc*, which show a diversity of dominant 3′ UTR isoforms in different adult tissues, across the embryonic timecourse, and in different cell lines (Fig. 2c; Additional file 1: Figure S4a).

Some genes exhibit double-digit numbers of termini. An extreme example is *Hrb27C*, for which our previous northern blotting analysis indicated at least seven distinct well-accumulated 3′ end isoforms [6], but for which aggregate 3′-seq datasets suggest dozens of distinct functional pA sites across its 6.7 kb 3′ UTR (Fig. 2d). Nevertheless, not all genes that express long 3′ UTRs express large numbers of discrete 3′ UTR isoforms. For example, *2mit*, *Vglut*, and *corin* are well-expressed genes with long (3–4 kb) 3′ UTRs that nevertheless all exhibit precise, single 3′-seq signals in heads (Fig. 2e; Additional file 1: Figure S4b).

The low level events, such as the majority of annotated ends in *Hrb27C*, retain features of bona fide PAS. Of note, 36% of ends that represent less than 5% of total gene expression still present one of the top three PAS types and present characteristic sequence composition around the cleavage site (Additional file 1: Figure S5), indicative of bona fide ends. This provides evidence that even lower-expressed 3′-seq termini are biochemically valid events generated through recognition of strong PAS. However, to provide a more practical measure of APA in *Drosophila*, we also segregate annotations of 3′ ends that comprise at least 5% of total expression of each locus (Additional file 6: Table S4; Additional file 7). This allows researchers to focus on isoforms that are more highly expressed and may be more likely to be empirically validated.

Overall, these genomic observations and individual gene examples illustrate that these 3′ UTR annotations vastly expand the complexity of terminal 3′ UTR isoform expression in *Drosophila*, and supply a key resource for the *Drosophila* community.

### Expanding the repertoire of tissue-specific 3′ UTR utilization in *D. melanogaster*

Several of the gene examples shown thus far illustrate robust tissue heterogeneity in deployment of 3′ UTR isoforms. Indeed, we previously documented that ~ 400 *Drosophila* genes express isoforms with long 3′ UTRs in the nervous system while ~ 100 genes express specific short 3′ UTR variants in testis, with intermediate isoforms in ovary [6]. However, our earlier studies could only document substantial APA differences evident from RNA-seq data between tissues, or occasionally as large expression change points within an individual library [16]. In fact, in northern blot analyses, we have noted the existence of intermediate 3′ UTR isoforms with clear neural bias, but that were not necessarily exclusive to the nervous system [6]; these would not have been discernible from RNA-seq data. Thus, the availability of 3′-seq data allowed us to examine the scope of tissue-specific APA patterns more broadly. The *Hairless* gene shows how 3′-seq clearly illustrates this characteristic pattern of tissue-specific APA isoforms, even though testis, ovary, and head each express multiple 3′ UTR isoforms for this locus (Fig. 2f); other examples are shown in Additional file 1: Figure S4.

We quantified 3′ isoform usage of genes between pairs of tissues by calculating a weighted 3′ UTR length metric, taken as the average of all 3′ UTR isoform lengths per gene weighted by the contribution of each isoform’s expression. Although we have annotated many very long (multi-kilobase) 3′ UTRs in *Drosophila*, the weighting scheme reduces the length score of the vast majority of loci to < 3 kb (Additional file 1: Figure S6). Therefore, we plot up to 3 kb to emphasize visualization of the data on a linear scale. By comparing the weighted length of 3′ UTRs that are co-expressed in testis and head (Fig. 2g), or co-expressed in ovary and head (Fig. 2h), we observe that > 1000 and > 700 genes (Additional file 8: Table S5) express longer isoforms in head with respect to either gonadal tissue, respectively. Strikingly, just a handful of genes were called as expressing shorter 3′ UTR isoforms in heads in either gonadal comparison. Upon comparing testis and ovary, we observe that the dominant signature is for genes to express shorter APA isoforms in testis (Fig. 2i).

Overall, these trends are consistent with our previous findings [6] that *Drosophila* testis expresses shorter 3′ UTRs and head expresses much longer 3′ UTRs. However, our 3′-seq data allow us to vastly expand the scope of the tissue-specific alternative 3′ UTR profiles, thereby emphasizing the regulatory impact of 3′ UTR isoform expression in *D. melanogaster*.

### Additional programs of 3′ UTR isoform expression in ovary and embryo timecourse

**Fig. 3:**
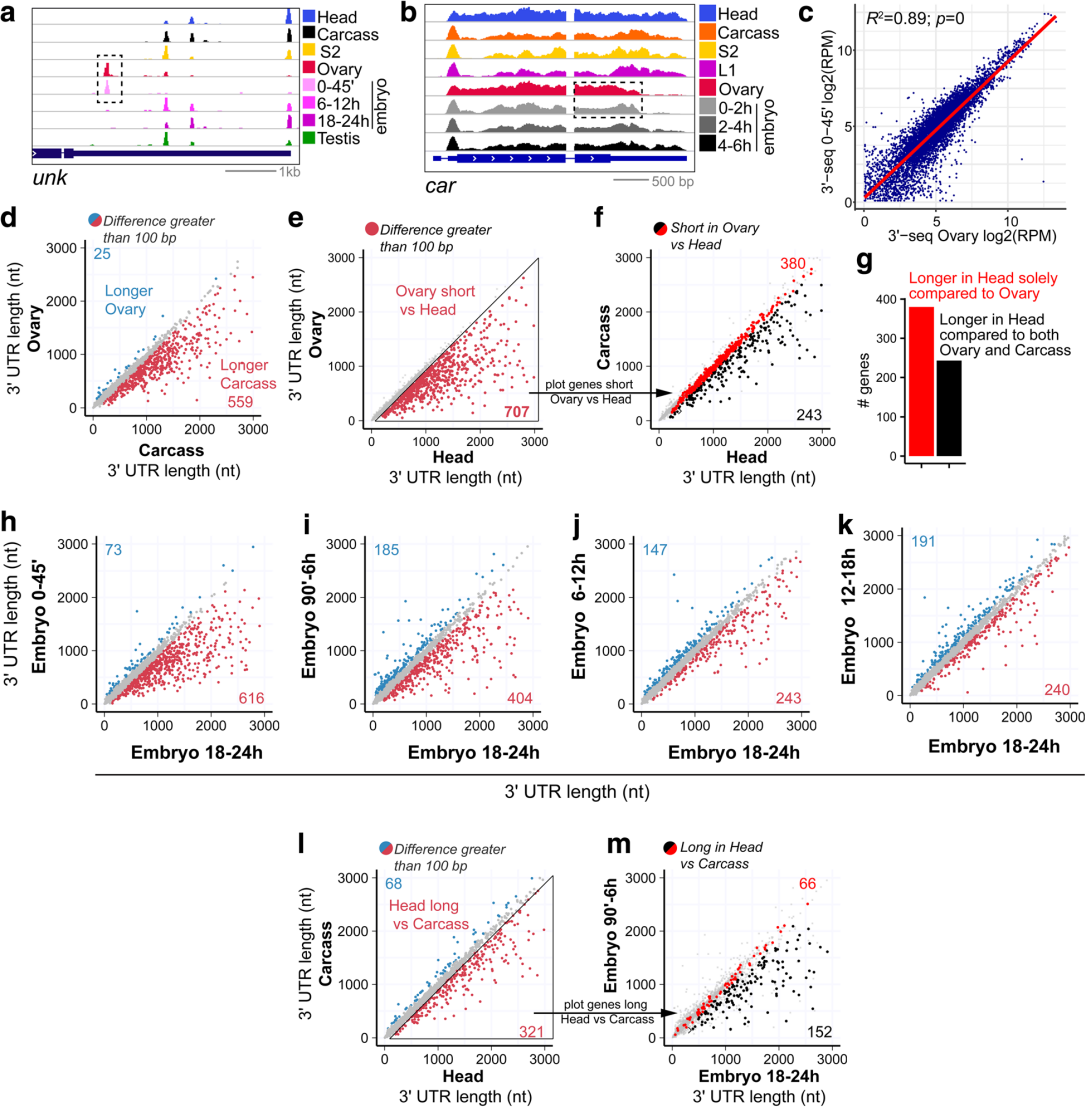
Specific patterns of APA in the ovary and across embryogenesis. **a** Examples of a gene that expresses a pattern of 3′ UTR isoform expression specific to the ovary and early 0–2 h embryo, compared to a range of other developmental stages, tissues, and cell lines. **b** RNA-seq data example of 3′ UTR shortening of a maternally deposited message. *Dotted line rectangle* shows common pattern of 3′ end isoform expression between ovary and early embryo. **c** Comparison of normalized 3′-seq data between ovary and very early 0–45-min embryos. Test of correlation with Spearman rank order rho and *p* value shown on top of the graph. **d**–**m** Weighted 3′ UTR length comparison between samples. Weighted 3′ UTR length is obtained taking the average of all 3′ UTR isoform lengths per gene weighted by the contribution of each isoform expression. Genes are expressed at a minimum of 5 RPM in all samples. Genes whose weighted 3′ UTR length differs by 100 bp or more between samples are colored, whereas all other loci are in *gray*. **d** Carcass expresses longer 3′ UTRs than ovary. **e** Head expresses longer 3′ UTRs than ovary. **f** When the subset of genes that is shorter in ovary vs head (from **e**, loci within *triangle*) is compared for length profile between carcass and head, we see that only a subset of them is extended in head. **g** Barplot summary of head vs. ovary and head vs. ovary + carcass 3′ UTR comparison. **h**–**k** Embryogenesis timecourse in which successive embryonic timepoints are plotted on the *y-axes* against the common, final embryonic timepoint on each *x-axis* (18–24 h). Across the timecourse of **h** 0–45′, **i** 90′–6 h, **j** 6–12 h, **k** 12–18 h, we see that progressively fewer genes are differentially shorter with respect to 18–24 h embryos. This reflects a 3′ UTR lengthening trend across embryogenesis. **l**, **m** Head lengthening and embryonic lengthening reflect a similar gene signature. **l** Defining the genes that are lengthened in head vs. carcass. **m** When these genes were overlaid onto the set that were co-expressed in both early and late embryos, we observe unilateral trend that they exhibit longer 3′ UTRs in 18–24 h embryos compared to 90′–6 h embryos

As mentioned, ovary generally expresses intermediate-length 3′ UTR isoforms relative to head and testis [6]. However, with our expanded and quantitative views, we realized there was a substantial population of genes whose dominant testis APA isoform was longer than in ovary (Fig. 2i). By browsing the 3′-seq data, we confirmed many loci, such as *unk*, *Su(dx)*, *car*, and *sina*, that express discrete shorter length 3′ UTR isoforms relative to other somatic or cell line samples (Fig. 3a; Additional file 1: Figure S7a). Moreover, such genes express the same short 3′ UTRs in very early (0–45′ and 45–90′) embryos, but they subsequently expressed longer 3′ UTRs upon onset of zygotic transcription. The zygotic 3′ UTR lengthening for such “ovary/maternal-short” genes was also evident in RNA-seq data (Fig. 3b) and confirmed by 3′-seq (Additional file 1: Figure S7a).

These observations led us to infer that a distinct gene set carries a characteristic 3′ UTR signature in ovary and very early embryos. Since the ovary is mostly comprised of developing eggs, the ovarian transcriptome is expected to be dominated by the maternal gene expression program. Consistent with this, the correlation of 3′-seq expression between ovary and 0–45′ embryo datasets is very high (*R* ^2^ = 0.89; Fig. 3c).

We therefore sought to identify 3′ terminal events that are specific to ovary/early embryos. To this end, we compared ovary data to the somatic male carcass (Fig. 3d), as well as to cell lines (Additional file 1: Figure S7), to identify specific “ovary-short” 3′ UTRs. We note the caveat that the carcass contains the ventral nerve cord and certain peripheral neurons, but cell lines should be devoid of mature neuron signatures. These comparisons change our perspective on tissue-specific APA. Many genes that we earlier classified as head-extended (Fig. 3e) are not extended with respect to carcass (Fig. 3f, red subset), although there certainly remain many hundreds of neural-extended loci. Instead, nearly twice as many loci proved to shorten their 3′ UTRs in ovary relative to carcass (Fig. 3d) than lengthen in head compared to carcass (Fig. 3f).

We performed similar comparisons of ovary and head to several cell lines and obtained a comparable picture (Additional file 1: Figure S7). Together, these analyses revealed > 400 genes in common that expressed shorter 3′ UTR isoforms in ovary relative to both male carcass and S2 cells. Similarly, we identify shortening events that are specific to testis by comparing 3′ UTR isoform expression in this tissue against female carcass or S2 cells. We report the list of genes that specifically shorten in gonads in Additional file 9: Table S6. Overall, our expanded 3′-seq atlas allowed us to appreciate that gonads exhibit independent trends of 3′ UTR shortening in *Drosophila*, and that a proportion of “head-extended” genes that we earlier characterized actually reflect ovary 3′ UTR shortening. Indeed, if we overlay onto this analysis the set of genes that we observed to express longer 3′ UTRs in head compared to ovary (Fig. 3e), we detect only 243 genes (Fig. 3f; Additional file 10: Table S7) expressing longer 3′ UTRs in head compared to both ovary and carcass (Fig. 3g). This confirms that a subset of the genes express specifically shorter 3′ UTRs in the ovary. Overall, these analyses define the maternal transcriptome as a novel, broad, tissue-specific APA setting in *Drosophila*.

We further interrogated the embryonic timecourse 3′-seq data. When examining modENCODE RNA-seq data that densely tile at 2-h intervals across 24 h of embryogenesis, we appreciated many loci that exhibited multiple tiers of progressive 3′ UTR extensions across development. The gene *shep* illustrates this phenomenon, whereby it starts with a short 3′ UTR in maternally deposited isoforms, expresses a longer 3′ UTR isoform at the start of zygotic transcription, and a further distal extension by late embryogenesis (Additional file 1: Figure S4D). This apparent developmental lengthening was a global phenomenon, and was not simply due to the “maternal short” program. Pairwise comparisons of all embryonic 3′-seq libraries showed that 3′ UTRs tend to lengthen progressively, even following zygotic transcription (Additional file 1: Figure S8). This can be easily visualized in a few pairwise comparisons of earlier embryonic libraries against the last timepoint (18–24 h). One can see that in the maternal transcriptome (0–45′; Fig. 3h), the dominant signature is for 3′ UTR shortening, with > 600 genes expressing a proximal isoform relative to 18–24 h embryos and only 73 genes expressing a distal isoform (Additional file 11: Table S8). In the comparison with 90′–6 h embryos (Fig. 3i), the bias is only half as strong, and in the next time point analyzed (6–12 h; Fig. 3j) it is weaker still; 12–18 h embryos are relatively similar in their 3′ UTR profiles as 18–24 h embryos (Fig. 3k).

Previous in situ hybridization experiments indicated that developmental embryonic 3′ UTR lengthening was due to selective expression of longer 3′ UTR isoforms in the embryonic central nervous system [6, 17]. To test if this was the basis of this phenomenon, we compared the “head lengthened” and “late embryo lengthened” gene sets. To do so, we first identified 321 genes whose 3′ UTRs were specifically extended in head compared to carcass (Additional file 12: Table S9), reflecting neural extension (as opposed to gonadal shortening, Fig. 3l). We then analyzed the behavior of the genes in this set that were also co-expressed in both 90′–6 h and 18–24 h embryo timepoints (218 genes). Of these, there was a clear unidirectional trend in which the only 3′ UTR differential genes were essentially all longer in 18–24 h 3′-seq library (152 genes; Fig. 3m; Additional file 13: Table S10). Thus, these data show that the apparent developmental lengthening of 3′ UTRs during embryogenesis includes a substantial component of tissue-specific extension of 3′ UTRs in the nervous system.

In summary, we demonstrate additional tissue and temporal APA patterns in our 3′-seq atlas, and reveal tissue-specific bases for apparent developmental shifts in 3′ UTR patterns.

### Conservation of tissue-specific APA across Drosophilid species

We next sought to assess the breadth of conservation and divergence of tissue-specific 3′ UTR isoforms. Despite the availability of a dozen fly genomes for nearly a decade, experimental evidence for their transcriptomes has been sparse, and their gene annotations have relied mostly upon comparisons with *D. melanogaster*. Only recently have substantial amounts of RNA-seq data begun to be available for different sequenced Drosophilids [37]. However, the bulk of these data were from whole animals, thus precluding assessment of tissue-specific isoforms.

We therefore analyzed close and distant relatives of *D. melanogaster*, namely *D. yakuba* and *D. virilis*, which are ~ 12 and ~ 62 million years diverged, respectively [38]. We generated companion 3′-seq and total RNA-seq libraries of head, ovary and testis for these species, and supplemented these with 3′-seq datasets across embryogenesis (Additional file 2: Table S1). We subjected these to the same pipeline for calling 3′ termini as with *D. melanogaster* and confirmed that the ends constitute high quality termini as seen by the nucleotide distribution surrounding the derived cleavage sites (Additional file 1: Figure S9). Of note, both species recapitulate insect-specific features we noted for *D. melanogaster* pA sites, such as the very proximal A-rich region (Fig. 1b) and the propensity for usage of the AAUAUA variant (as analyzed in later figures).

**Fig. 4:**
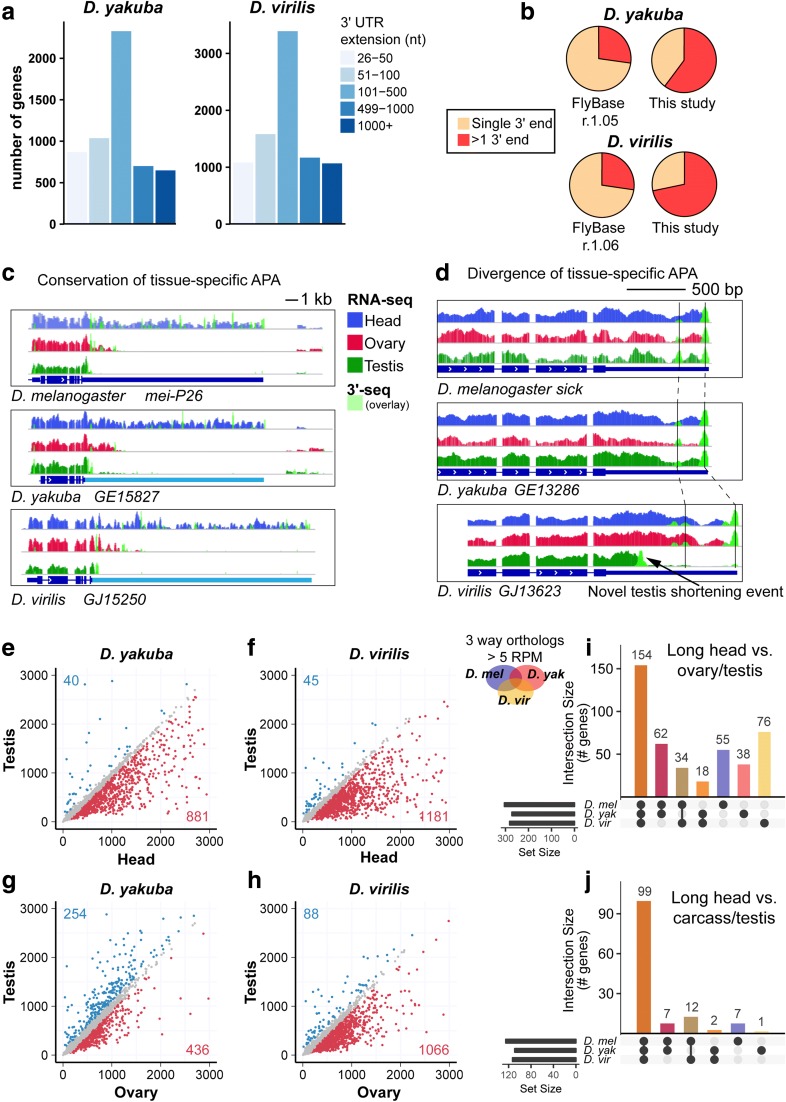
Evolution of-tissue specific APA across *Drosophila* species. **a** Reannotation of *D. yakuba* and *D. virilis* 3′ UTRs from deep 3′-seq data yields thousands of 3′ UTR extensions. **b** Percentage of genes with either one annotated end or more than one for the current *D. yakuba* annotation (r1.05) or *D. virilis* annotation (r1.06) and our revised 3′-seq-based atlas. **c**, **d** Examples of expression of genes with orthologs in *D. melanogaster*, *D. yakuba*, and *D. virilis*. RNA-seq and 3′-seq of head, ovary, and testis are shown as illustrated in the legend. 3′-Seq is overlaid in *light green* onto the RNA-seq track. **c** Example of a gene with conserved tissue-specific patterns of 3′ UTR expression. Extension of the annotation of *D. yakuba* and *D. virilis* is shown in *light blue*. **d** Example of a gene that shows de novo expression of a dominant short 3′ UTR isoform in the testis of *D. virilis*. **e**–**h** Weighted 3′ UTR length comparison between tissues. Weighted 3′ UTR length is obtained taking the average of all 3′ UTR isoform lengths per gene weighted by the contribution of each isoform expression. Genes are expressed at a minimum of 5 RPM in all samples. The genes for which weighted 3′ UTR length differs by 100 bp or more between samples are shown colored: *red*, longer weighted length in the sample on the *x-axis*; *blue*, longer weighted length in the sample on the *y-axis*. Comparisons are shown for *D. yakuba* and *D. virilis*. **e**, **f** Head vs testis. **g**, **h** Testis vs ovary. **i** Conservation of tissue-specific APA. The overlap of the genes that express a weighted 3′ UTR length of more than 100 bp in head compared to both testis and ovary, have orthologs in all three species, and are expressed at least at 5 RPM was taken and is represented as a bar graph. Schematic Venn diagram and dots below the bar graph show the nature of each intersection. Horizontal bar graph shows the number of genes considered for each species. **j** Preferred conservation of head extensions across Drosophilid phylogeny. This analysis was done as in **i**, except that the gene set was first conditioned on genes with specific evidence for head-extension in *D. melanogaster* from comparison to carcass (e.g., Fig. 3l), then were overlaid onto the gene sets with differential 3′ UTRs between head and testis 3′-seq datasets across species. Nearly all of these loci exhibit a conserved pattern

These efforts allowed us to significantly improve the 3′ end annotation of these species. Since there has been comparatively little experimental annotation of 3′ termini in these other species, the improvements in the breadth and refinement are much more substantial than in *D. melanogaster*. We provide catalogs of 36,864 pA sites in *D. yakuba* (Additional file 3: Table S2; Additional file 14) and 53,579 pA sites in *D. virilis* (Additional file 3: Table S2; Additional file 15), extending the 3′ UTR space of 5581 protein-coding genes by 2.4 Mb in *D. yakuba* and 8293 genes by 8.9 Mb in *D. virilis* (Fig. 4a; Additional file 5: Table S3). In sum, these data allow us to increase the number of genes that undergo APA from one quarter to ~ 60% in *D. yakuba* and ~ 75% in *D. virilis* (Fig. 4b).

Browsing of individual loci provided striking confirmation of the conservation of individual cases of neural-specific 3′ UTR lengthening and testis-specific 3′ UTR shortening that we had characterized in *D. melanogaster* [6]. For example, the longest 3′ UTR we had experimentally validated was the 18.5 kb 3′ UTR of *mei-P26* that is expressed in *D. melanogaster* nervous system, including adult heads. Its predominant isoforms are intermediate in ovary and short in testis. We observe these same tissue-specific APA trends in the other species, both in total RNA-seq and in 3′-seq data (Fig. 4c). Consistent with their phylogenetic relationships, the *D. melanogaster* isoforms are more similar to *D. yakuba* than to *D. virilis*. Notably, the *D. virilis mei-P26* 3′ UTR has expanded to 22 kb, making it one of the very longest 3′ UTRs known in an invertebrate. Perhaps unsurprisingly, these long 3′ UTRs are misannotated in public databases. On the other hand, we also observe evolutionary novelty in 3′ UTR isoforms. For example, for the *sick* locus, *D. virilis* retains the same major pA sites used in head and ovary as its *D. melanogaster* and *D. yakuba* orthologs, but it uses a novel dominant proximal pA site in testis (Fig. 4d).

When we evaluated *D. yakuba* and *D. virilis* at the genome level, we observed that both species recapitulated very strong trends to express globally longer 3′ UTR isoforms in head compared to testis (Fig. 4e, f), and globally longer 3′ UTR isoforms in ovary compared to testis (Fig. 4g, h). As mentioned, since we document a signature of maternal shortened transcripts, this appears to manifest in the fact that the length bias between head and testis is much more unidirectional in all three species, whereas there is a population of transcripts with bias to express longer 3′ UTR isoforms in testis relative to ovary. This was more so the case in *D. yakuba* than in *D. virilis*.

Overall, these experimental samplings across different branches of the *Drosophila* phylogeny clearly demonstrate that tissue-specific APA patterns are preserved across fruit fly evolution. However, it does not necessarily reflect whether the same genes are subject to tissue-specific APA. To test this, we compared the weighted 3′ UTR length between tissues in pairs of species. Note that this calculation assesses whether tissue-characteristic expression of 3′ UTRs is conserved, but does not take into account if the sequences of pA sites themselves are conserved (this is addressed later). In order to be more certain about APA calls for individual genes, we restricted this analysis to genes for which orthologs were called in all three species and expressed at > 5 RPM in all three tissues.

When we conditioned this analysis for genes that expressed longer 3′ UTRs in head vs. both ovary and testis, we found that 154 genes conserved this property across all three species (Fig. 4i; Additional file 16: Table S11), with a subset of genes retaining this in two out of three species. This indicates that length differences between head and gonads are generally under strong constraint across the Drosophilid phylogeny. Nevertheless, there are a substantial number of species-specific cases in which the head isoform is longer than gonadal isoforms (Fig. 4i; Additional file 16: Table S11).

To delve further, we conditioned the analysis on *D. melanogaster* genes for which we have auxiliary evidence for specific head-lengthening, based on comparison to carcass (Fig. 3l). We then asked what set of these genes were also lengthened across fly species when comparing head vs testis. In this analysis, we obtain a very different outcome in which the vast majority of loci are conserved in their tissue-specific APA profile (Fig. 4j; Additional file 17: Table S12). We conclude from this that neural 3′ UTR extension is under strong preferential conservation across species, but many other instances of apparent species-specific differences where head isoforms are longer than gonadal isoforms actually reflect de novo gonadal 3′ UTR shortening events. Indeed, such events can be seen by inspection of the 3′-seq and RNA-seq data (e.g., Fig. 4d).

Overall, the substantial evolutionary conservation of tissue-specific APA profiles indicates that these post-transcriptional processes incorporate into necessary regulatory regimes. On the other hand, the evolutionary plasticity of tissue-specific APA provides us an opportunity to examine the underlying genomic changes that can alter 3′ isoform deployment. We explore this in a later part of this study.

### Expanding annotations of deeply conserved miRNA and RNA binding protein sites

Although new miRNAs continue to be identified all the time, the vast majority of these cloned over the past decade are species-specific loci that are poorly expressed, and whose regulatory impact is therefore unclear. The catalog of well-conserved miRNAs, which are believed to be the major effectors of the miRNA network, has not changed much during this time save for a handful of atypical loci. There continue to be active discussions on how best to predict their breadth of possible species-specific targets, or non-canonical target sites, or unconventional transcript targets [39–41], but the overall tenet remains that an initial go-to list of sites comprise well-conserved seed matches to well-conserved miRNAs, especially sites that reside within locally conserved regions [42, 43].

However, underlying such predictions is a comprehensive set of 3′ UTR annotations. For example, the popular TargetScanFly v6.2 resource (http://www.targetscan.org/fly_12/) utilizes 3′ UTR annotations from over 5 years ago which lack substantial genuine 3′ UTRs, including many neural 3′ UTR extensions. We therefore utilized the TargetScanS algorithm to perform de novo predictions of target sites for deeply conserved *Drosophila* miRNAs using our 3′ UTR annotations. Individual researchers must choose for themselves at what level of stringency they wish to utilize predictions, deciding on a balance of stringency to reduce false positives and leniency to cast a wider net for candidates. However, we highlight some statistics at various levels of site conservation using the 3′ UTR annotations used in TargetScanFly v6.2, as well as FlyBase r6.12 and by our newest extensions.

**Fig. 5:**
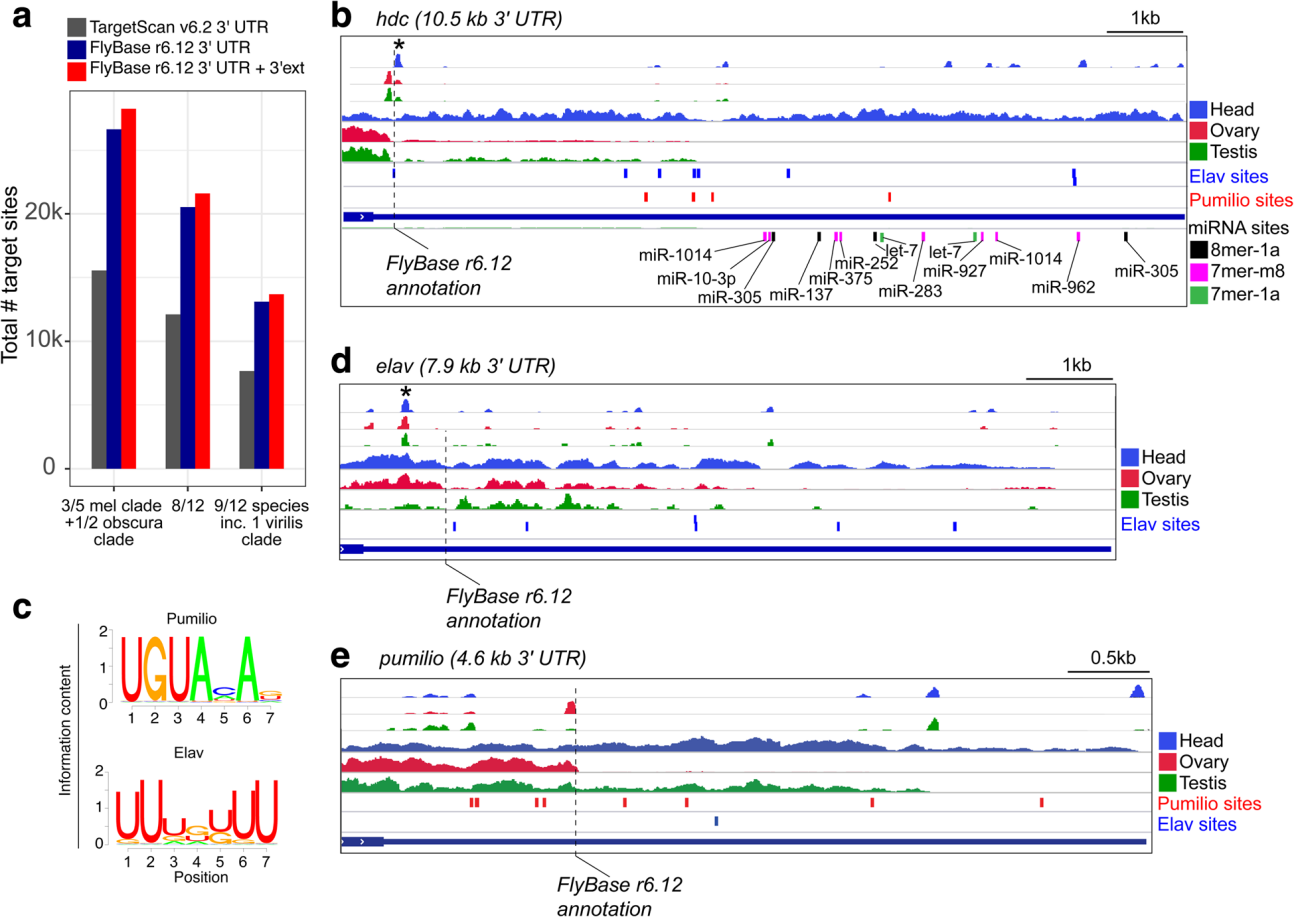
Annotation of well-conserved miRNA, Elav, and Pumilio binding sites. **a** We used TargetScanS algorithm to perform target predictions using the 3′ UTR databases used in the current available releases of TargetScanFly (v6.2), FlyBase (r6.12), and our current 3′ UTR annotations based on 3′-seq atlas (FlyBase + ext). This graph reports only 7mer-m8 seed matches to pan-Drosophilid conserved miRNAs, bearing conservation properties across the indicated fly species. **b** Example of *headcase* (*hdc*). TargetScanFly (v6.2) annotates no miRNA binding sites in the short 3′ UTR; however, an abundance of deeply conserved miRNA, Elav, and Pumilio binding sites exists in the much longer 3′ UTR that is well-expressed in head. **c** Position weight matrices for Pumilio and Elav binding. **d** The *elav* 3′ UTR has an abundance of conserved Elav binding sites. **e** The *pumilio* 3′ UTR has an abundance of conserved Pumilio binding sites. RNA-seq and 3′-seq are shown for each gene example. *Asterisks* denote likely false positive 3′-seq signal deriving from internal priming of genomically templated A-rich sequence

We utilized only canonical 7mer-m8 seed matches for this search, although we subdivided those that have t1A pairing in the annotation. When considering the most stringent level of conservation (see “Methods”), by requiring a site to be conserved in 9/12 fly species [38] including at least one virilis-clade species (*D.vir/D.moj/D.gri*), we predict 13,702 deeply conserved target sites located on 3700 3′ UTRs. This is ~ 80% more than TargetScanFly v6.2 and includes 589 more sites found on our newly annotated extensions (Fig. 5a; Additional file 1: Figure S10). Larger numbers of sites are predicted using less stringent conservation criteria. We illustrate the example of *headcase*, for which the 3′ UTR used for TargetScanFly v6.2 annotations is shown, but the dominant isoform expressed in head has a 10.5-kb 3′ UTR. This long 3′ UTR contains numerous deeply conserved 7mer-m8/t1A seed matches for various miRNAs (Fig. 5b; Additional file 1: Figure S10) and additional deeply conserved 7mer-1a seed matches (even though this weaker site type was not included in the tallies in Fig. 5a). These observations highlight the fact that using popular online resources can severely underestimate the breadth of highly conserved miRNA target networks. We provide these annotations of miRNA sites in Additional file 18: Table S13 as well as in GTF format files (Additional file 19).

Since we previously noted that Pumilio and Elav binding sites appeared to be enriched in neural 3′ UTR extensions [6], we also annotated well-conserved matches to binding motifs for these RNA binding proteins (RBPs; Fig. 5c). At the deepest level of conservation, we identify 1978 and 1045 conserved binding sites for Pumilio (UGUANAN) and Elav (UUUGUUU), respectively. Indeed, *headcase* contains many well-conserved sites for both Elav and Pumilio (Fig. 5b). We also identify likely roles for autoregulation of these RBPs. We recently re-annotated the extent of the *elav* 3′ UTR [44], and we find a multitude of conserved Elav binding sites on common and alternative portions of the *elav* 3′ UTR, although none for Pumilio (Fig. 5d), in line with the known role of Elav in regulating its own 3′ UTR [45]. Reciprocally, the *pumilio* 3′ UTR contains an abundance of deeply conserved binding sites for Pumilio, but only one for Elav (Fig. 5e). The annotations of conserved Pumilio and Elav binding sites are provided in Additional file 20: Table S14 as well as in GTF format files (Additional file 21).

### Impact of PAS identity on alternative pA site usage

The efficiency of cleavage and polyadenylation is affected by the strength of the underlying PAS [46, 47]. For example, proximal bypassed pAs found in 3′ UTRs generally exhibit weaker PAS, as evidenced by a lower percentage of such sites bearing a canonical AAUAAA PAS [34, 48]. To assess trends of *Drosophila* PAS strength observed from previous studies of small sets of EST-derived ends [32], we searched for differences in PAS identity in the 50-nucleotide windows upstream of our atlas of pA sites. We divided sites according to their residence in genes according to single vs multiple ends, and according to their tissue-specific APA properties.

**Fig. 6:**
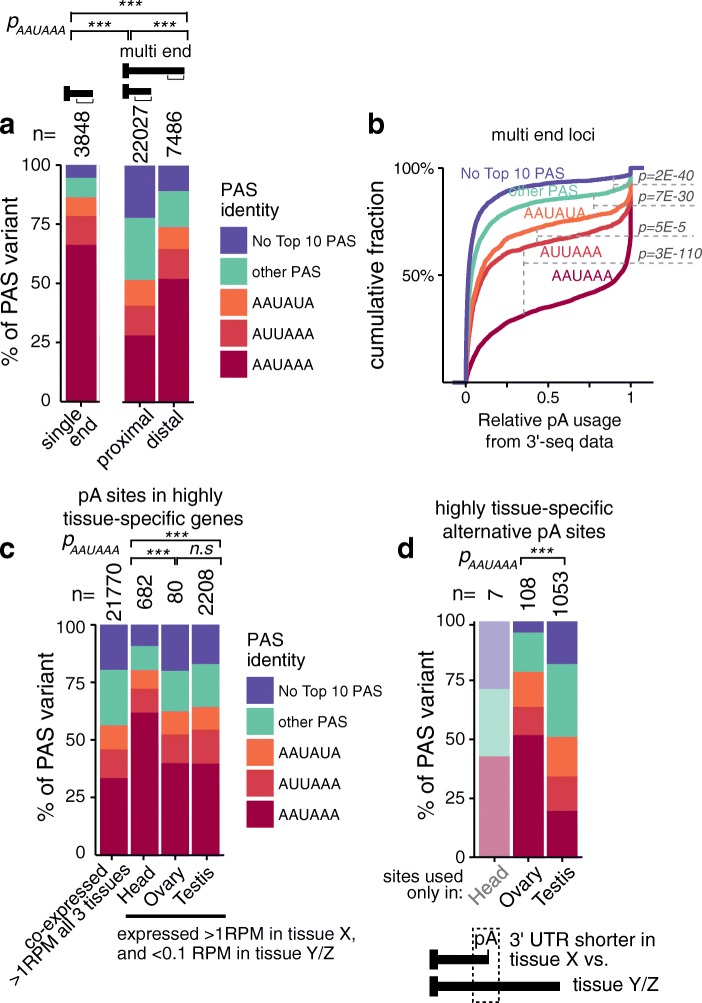
Distinctive properties of alternative and tissue-specific poly(A) sites. **a** Percentages of PAS types present in the 50-nucleotide window upstream of the pA site. pA sites were divided based on 3′ UTR class and position. *Single end*, pA sites that define the 3′ end of genes with only evidence of single terminal 3′ UTR isoform; *proximal*, pA sites found in the 3′ UTR of genes that undergo APA, all pA sites upstream of the most distal one; *distal*, the last pA sites of terminal 3′ UTRs that undergo APA. Proximal sites of APA genes exhibit distinctively lower fraction of optimal PAS. **b** Empirical cumulative distribution functions (ECDF) of the relative strength of all pA sites in terminal 3′ UTRs subdivided by the identity of the PAS (as shown in the legend on the *left*) in the 50 nucleotides upstream of the cleavage site. The relative strength score represents the fraction of isoforms ending at a specific site compared to downstream sites. This was calculated from the fraction of reads at each pA out of all reads at that site or downstream ones. The relative pA site usage of each PAS variant class compartmentalizes well according to their known efficiency of cleavage. The significant differences of the relative strength score among PAS identities were calculated by Wilcoxon rank-sum tests. **c** Analysis of PAS identity within tissue-specific genes. We compared genes that were co-expressed in the three tissues (> 1 RPM in head, ovary, and testis) vs ones that were deemed tissue-specific (> 1 RPM in one of these tissues, < 0.1 RPM in the other two). All PAS in these tissue-specific genes were plotted. The head utilizes a distinctively elevated fraction of optimal PAS. **d** Analysis of PAS identity at tissue-dominant pA sites. Percentages of PAS types that present in the 50-nucleotide window upstream of the pA site. pAs were divided based on tissue dominance in either head (*n* = 7), ovary (*n* = 108), or testis (*n* = 1053). Data for the head are opaque since so few available sites are head-specific. Testis APA sites utilize a broader range of suboptimal PAS compared to ovary APA sites. *** *p* < 0.0005; n.s. *p* > 0.05

When we consider pA sites of single end genes, we observe strong over-representation of the canonical PAS (66%; Fig. 6a). Amongst the terminal pA sites of 3′ UTRs that undergo APA, we also observe similar enrichment of stronger PAS, although to a lesser extent (34%, *p* = 2E-312, Fisher’s exact test). However, proximal alternative pA sites distinctly carried a lower proportion of canonical PAS compared to distal pA sites (28 vs 52%, *p* = 1E-298), with higher frequencies of weak variants (Fig. 6a). This confirms our previous finding of reduced levels of canonical AAUAAA upstream of “bypassable” pA sites in *Drosophila* [6] and the general understanding that proximal pA sites found in 3′ UTRs are associated with weaker PAS [21]. We also evaluated these three classes of pA sites for differences in DSE composition. We observed that proximal pA sites specifically exhibited moderately weaker DSE enrichment than the other two classes (Additional file 1: Figure S11), consistent with the notion that this contributes to their capacity to be bypassed under certain conditions.

Recently, it was reported that RNA structures are more highly enriched in the space between the PAS and the cleavage site as a function of distance, playing a role in 3′ end processing by juxtaposing PAS and cleavage sites [49]. We analyzed the structure-forming potential between the canonical PAS AAUAAA and the resulting pA cleavage sites as a function of distance between the two elements. Indeed, we observed that pA cleavage sites that have AAUAAA PAS at a greater distance (> 30 nucleotides) showed more stable structure-forming potentials between PAS and cleavage site compared to AAUAAA PAS which were more proximal to the cleavage site (< 30 nucleotides) (*p* < 0.04). pA events that lack any of the top ten PAS hexamers upstream of the cleavage site in a 100-nucleotide window show no structure-forming potentials compared to shuffled sequences (*p* < 1E-9; Additional file 1: Figure S12). This supports the concept that structured elements might bring PAS that are further away from the cleavage site (> 30 nucleotides) into closer proximity to sites of endonucleolytic cleavage to mediate effective 3′ end formation.

To test for correlation between PAS identity and 3′ UTR isoform expression, we determined the relative strength score at each pA site, which represents the fraction of isoforms ending at a specific site compared to downstream sites. This was calculated from the fraction of reads at each pA site out of all reads at that site or downstream. When we look at the cumulative relative strength distribution of pAs according to PAS, we observe that known stronger PAS variants correlate well with their relative functional usage (Fig. 6b). Indeed, the PAS variants separate completely in their CDF profiles as AAUAAA > AUUAAA > AAUAUA > other common PAS variants > no common PAS variants. These findings are remarkable considering there are many other post-transcriptional regulatory reasons that could determine the final steady-state levels of a given transcript observed in cells [50], yet core PAS identity proved to have a strong observable impact.

Overall, these results confirm that, in *Drosophila*, as in other eukaryotes, core 3′ end formation sequences and structures likely contribute to generation of alternative 3′ end isoforms and PAS identity contributes to the final relative steady state levels observed for individual isoforms.

### Impact of PAS identity on tissue-specific pA site usage

We next examined if there were differences in pA sites of tissue-specific termini. These can exist in genes that are inherently tissue-specifically expressed, or that are more broadly expressed but undergo tissue-specific APA. To start, we isolated genes that were specific to head, ovary, or testis (i.e., expressed in one of these tissues at > 1 RPM and < 0.1 RPM in the two companion tissues) and compared these to broadly expressed genes (> 1 RPM in all three tissues). When we plotted PAS frequencies amongst the pA sites in these cohorts, we specifically observed increased usage of canonical AAUAAA amongst head-specific genes (Fig. 6c) (head, 62%; ovary, 40%; testis, 40%; *p*_H vs O_ = 2.6e-4, *p*_H vs T_ = 3.2E-24, *p*_O vs T_ = 1, Fisher’s exact test). A higher stringency in pA site definition in head would be consistent with a propensity to express longer 3′ UTR isoforms in the nervous system by skipping suboptimal pA sites.

Given that the strength of the PAS can influence the relative levels of 3′ UTR isoform accumulation [46, 47, 51], we asked if there was a correlation between tissue-specific 3′ UTR isoform expression and putative pA strength. To do so, we identified cohorts of tissue dominant pA sites, defined as those with at least 30% higher relative strength in one tissue compared to the other two tissues, based on strength scores derived for each tissue. There were barely any head-dominant pA sites (7), consistent with prior observations that head predominantly undergoes lengthening (Figs. 2 and 4), and may have more stringent rules for pA site recognition (Fig. 6c). On the other hand, we identify a much greater number of testis-dominant pA sites (1053) compared to ovary (108), suggesting that testis has idiosyncratic rules for PAS recognition. When we look at the PAS upstream of these tissue-specific pA sites, we notice that the dominant ends in testis have much less canonical AAUAAA PAS usage compared to ovary (20 vs 52%, *p* = 3E-12; Fig. 6d). These data provide evidence that the *Drosophila* testis has relaxed rules for recognition of pA sites.

### Sequence divergence in *cis*-regulatory motifs drive evolutionary changes in APA

**Fig. 7:**
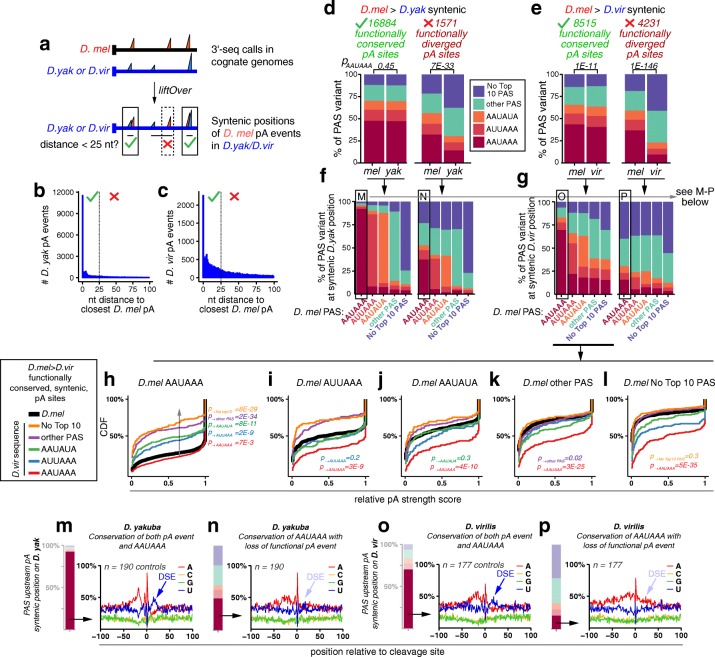
Evolutionary divergence of core poly(A) motifs has strong impact on poly(A) site usage. **a** The pipeline for conservation analysis. *D. melanogaster* pA sites were taken and syntenic locations on the genome of the species in the comparison were obtained using liftOver. *D. melanogaster* pA sites were considered functionally conserved if the syntenic position was within 25 bp of a 3′-seq annotated 3′ end in the compared species. **b**, **c** Numbers of *D. melanogaster* functional pA sites annotated to their closest functional pA sites in *D. yakuba* (**b**) or *D. virilis* (**c**). The most frequent occurrence is for precise synteny of functionally called pA sites at the nucleotide level. **d** Comparison of *D. melanogaster* and *D. yakuba* sites. Amongst functionally conserved sites, the distribution of PAS identities is similar in the two species. Amongst functionally divergent sites, where the syntenic position in *D. yakuba* is no longer a functional pA site, the PAS quality deteriorates. **e** Comparison of *D. melanogaster* and *D. virilis* sites. Similar trends are observed as above. **f** Comparison of *D. melanogaster* and *D. yakuba* sites, subdivided by PAS type. The pA site in *D. melanogaster* is noted below, and the *bar* represents the distribution of syntenic PAS sites in *D. yakuba*. The PAS site types are color coded as above. **g** Comparison of *D. melanogaster* and *D. virilis* sites, subdivided by PAS type. In **f** and **g**, the sites boxed and labeled *M–P* are analyzed in the corresponding panels below. **h**–**l** Relative strength of pA sites change according to change in PAS identity during evolution. These data were plotted as empirical cumulative distribution functions of the relative strength in head of conserved pA sites between *D. melanogaster* and *D. virilis*, i.e., the sites take from the left set of panels in **g**. Different graphs are subsets of conserved pA sites according to the identity of the original *D. melanogaster* PAS. The relative strength distribution of the original pAs in *D. melanogaster* is shown as a *thick black line*. Relative strength of the conserved pA sites in *D. virilis* are plotted according to PAS identity of conserved pAs in that species (*colored lines*). At functionally conserved, syntenic, pA sites, specific changes in PAS identity correlate perfectly with changes in the relative pA site usage, depending on whether the site has become stronger or weaker during evolution. The significant differences of the relative strength score between *D. melanogaster* PAS and converted *D. virilis* identities were calculated by Wilcoxon rank-sum tests. **m**–**p** Loss of a U-rich DSE is associated with loss of cleavage and polyadenylation at syntenic sites with conserved AAUAAA PAS. Nucleotide distribution around syntenic positions of *D. melanogaster* pA sites in either *D. yakuba* (**m**, **n**) or *D. virilis* (**o**, **p**). **m** Control set of functionally conserved pA sites in *D. yakuba* with canonical AAUAAA exhibits the expected pattern of nucleotide bias surrounding cleavage sites, including a strong downstream sequence element (*DSE*, *blue arrow*). **n** Nonfunctional *D. yakuba* sites that are syntenic to functional *D. melanogaster* pA sites and retain AAUAAA lack DSEs. **o**, **p** Similar comparisons of control (**o**) and nonfunctional *D. virilis* sites that are syntenic to functional *D. melanogaster* pA sites and retain AAUAAA (**p**) show loss of DSE in the latter. The barplots at the left of **m**–**p** indicate the PAS variant fractions; the AAUAAA type was used only for this analysis

Given the observed patterns of conservation of 3′ UTR isoform expression (Fig. 4), we investigated pA site evolution by leveraging pair-wise sequence alignments. We took advantage of the liftOver tool developed by the UCSC Genome Browser group (https://genome.ucsc.edu/cgi-bin/hgLiftOver) to identify syntenic positions of *D. melanogaster* 3′ ends of terminal 3′ UTR isoforms in either the *D. yakuba* or *D. virilis* genomes. We defined a 3′ end event as functionally conserved if we could find an orthologous event in the other species within 25 nucleotides (Fig. 7a). Of 35,986 sites falling in terminal 3′ UTRs, 16,884 were functionally conserved in *D. yakuba* (48%) while 8515 were conserved in *D. virilis* (22%). It is worth noting that 68% of functionally conserved events between *D. melanogaster* and *D. yakuba*, are positionally at exactly the same nucleotide (Fig. 7b), while 26% of sites exhibit nucleotide-level syntenic conservation out to *D. virilis* (Fig. 7c). These relative proportions correlate with the relative evolutionary distances amongst these species. Our ability to map such large numbers of functionally conserved sites to the nucleotide emphasizes the precision with which our subsequent evolutionary analysis is based upon, and also serves to validate our overall pipeline for calling 3′ end events from deep sequencing data.

We next interrogated the degree to which PAS motifs were preserved at functionally conserved pA sites. To do so, we compared the PAS upstream of *D. melanogaster* pA events to the PAS upstream of the syntenic position in the other species. The overall distribution of PAS sequence variants was similar upstream of the collection of syntenic, functionally conserved events between *D. melanogaster* and both *D. yakuba* (for canonical AAUAAA, 48 vs 48%, *p* = 0.45, Fisher’s exact test) and *D. virilis* (46 vs 41%, *p* = 3.9E-11), whereas there was substantial divergence in *D. yakuba* (32 vs 14%, *p* = 7.1E-33) and *D. virilis* (37 vs 9%, *p* = 1.1E-146) at the respective locations of pA sites that had lost functionality (Fig. 7d, e). To gain further insight, we examined the conservation of each subclass of PAS variant. In *D. yakuba*, we observe striking conservation of the exact PAS found upstream of the *D. melanogaster* site, for each class ranging from strong to weak variants (~90% for canonical AAUAAA and ~ 75% for weaker PAS) (Fig. 7f). In *D. virilis*, we only find the canonical AAUAAA unchanged at functionally conserved, syntenic positions ~ 70% of the time, while the weaker variants appear to be conserved at the sequence level only ~ 30% of the time. Curiously, we also observe a higher degree of weaker PAS converting to a stronger AAUAAA at these functionally conserved syntenic sites in *D. virilis* (22%) compared to *D. yakuba* (7%) (*p* = 7E-138; Fig. 7g).

Such a directional change might make sense if there was an accompanying change in expression usage of these ends, given that we have observed such a correlation within an individual species (Fig. 6b). We therefore tested if alteration in PAS quality during evolution can impact isoform usage, bearing in mind that, in each case, we are testing pairs of functionally conserved sites defined from experimental data in both species. To our knowledge, such an analysis has not been previously attempted at the genome scale. To do so, we analyzed the cumulative distribution of the relative strength of PAS that change in identity. We focused on the comparison between *D. melanogaster* and *D. virilis*, the most evolutionarily divergent pair, for which the largest number of evolving, yet functionally conserved, sites was available (sites from Fig. 7g, left panel set, replotted in Fig. 7h–l separated by PAS evolutionary change type).

We were pleased to observe very clear results in all comparisons, which reflected decrease or increase in relative pA site usage in accordance with evolutionary change in PAS strength (Fig. 7h–l). For example, for *D. melanogaster* pA sites that bear AAUAAA, but have a different variant at the syntenic position in *D. virilis*, the relative strength of those sites decreases in the same ordered fashion (Fig. 7h) expected as their overall relative site strength observed across all *D. melanogaster* pA sites (Fig. 6b). On the other hand, the weakest sites in *D. melanogaster* (lacking a top PAS) exhibit reciprocal behavior. Their functional counterparts in *D. virilis* exhibit stronger levels of recognition when their PAS is stronger (Fig. 7l)—e.g., when the corresponding PAS in *D. virilis* is AAUAAA (*p* = 5E-35)—while the *D. virilis* sites behave similarly to the ones in *D. melanogaster* when there is no top ten PAS (*p* = 0.3). All of the intermediate pA sites can be seen to evolve into correspondingly functionally weaker or stronger sites (Fig. 7i–k). These analyses reveal the importance of PAS identity in influencing the degree of pA site recognition in vivo and demonstrate that nucleotide-level changes in the PAS can drive measurable changes in APA isoform usage during evolution.

We also explored the conservation of other elements, such as the DSE. To this end, we were intrigued by a population of functionally divergent pA sites that, nonetheless, can be mapped to syntenic positions between *D. melanogaster* and other fly species and in particular retain the canonical AAUAAA PAS (Fig. 7f, g, boxed bars). What can explain the change in their utilization? We plotted the nucleotide composition surrounding these cleavage sites and compared them to equivalent numbers of control sites that are functionally conserved between the respective species and also retain AAUAAA. In *D. yakuba*, control conserved pA sites (Fig. 7m) mirror what we observed in *D. melanogaster* (Fig. 1b), that is, we observe the upstream A-rich sequence reflecting the PAS and the downstream U-rich sequence indicative of the DSE. However, the nucleotide composition at the *D. yakuba* sites that retain AAUAAA but are not functionally utilized is noticeably different, in that there is less evidence of DSEs (Fig. 7n). We repeated this analysis for *D. melanogaster* functional sites that are not recognized in *D. virilis* yet retain AAUAAA and obtained a similar view. Control *D. virilis* 3′ ends exhibit a prominent DSE (Fig. 7o), whereas locations that lack 3′ end evidence in the face of an optimal PAS clearly lack a DSE (Fig. 7p).

Overall, these species data comprise an enormous in vivo mutagenesis experiment, from which we have been able to use quantitative data from deep sequencing of 3′ ends to compile broad information on specific *cis*-alterations associated with evolutionary changes in alternative polyadenylation usage. Perhaps surprisingly, we document that a substantial amount of evolutionary divergence can be explained simply by specific divergence within PAS and/or DSE motifs. Although it is certainly the case that 3′ end formation is influenced by auxiliary RNA-binding proteins that influence the activity of the core polyadenylation CPA machinery, these data suggest that a major driver of evolutionary change in APA isoform usage is mediated directly through changing the efficiency of recognition by the CPA activity at a given pA site.

## Discussion

### Comprehensive atlases of 3′ UTR isoforms across multiple *Drosophila* species

Despite almost two decades of genome-wide efforts to annotate the *Drosophila* transcriptome, the 3′ ends of genes have eluded careful characterization [52]. This is the case despite recent reports of tissue-specific expression of specific 3′ end isoforms in *Drosophila* and RNA-seq based estimates of 3′ end isoform complexity that suggested that ~ 50% of genes express multiple 3′ end isoforms [6, 17]. Here, we deploy a 3′-seq protocol that we developed to generate a genome-wide atlas of sites of polyadenylation. We expand the number of genes that undergo APA to more than 65% of all genes, bringing *Drosophila* in line with what has been observed in other high eukaryotes [26]. We provide an atlas of 3′ end isoforms that covers 97% of all expressed genes, which we hope will be incorporated in a future annotation of *D. melanogaster*. Additionally, we generated 3′ end annotation of head, ovary, and testis, as well as time-points that cover all of embryonic development, for two additional species of *Drosophila*.

These combined datasets should be of general utility to both the *Drosophila* community as well as scientists interested in investigating aspects of the evolutionary conservation of 3′ UTR isoform expression and formation. We will work to incorporate our pA site annotations in public databases (e.g., UCSC Genome Browser, FlyBase) so that our revised gene models are broadly accessible. These data can have substantial impact for considering the post-transcriptional regulation of genes of interest. For example, we and others have shown how the realization of previously unannotated *Drosophila* neural 3′ UTR extensions helped elucidate critical miRNA biology, sometimes mediated via alternative aspects of 3′ UTRs [44, 53, 54]. The broadened catalogs of deeply conserved miRNA/RBP binding sites in *Drosophila* that we provide in this study via our updated 3′ UTR annotations can guide future studies of post-transcriptional regulation, especially in the nervous system. For those interested in the *Drosophila* genomics, our 3′-seq data can undoubtedly be mined in many additional ways. For example, while we focused this study on alternative 3′ UTRs of protein-coding genes, there are populations of intronic events (Fig. 1) that can be further interrogated to characterize intronic polyadenylation. In addition, there is another population of apparent intergenic events (Fig. 1) that presumably correspond to currently unannotated transcripts, and may include non-coding RNAs. Indeed, manual browsing indicates directional enrichment of RNA-seq signal upstream of many such intergenic events. In the future, these 3′-seq data can be integrated with other transcriptome and genome-wide datasets to further characterize RNA processing across *Drosophila* species.

It is important to note that 3′-seq data, as well as all data obtained using short read-based RNA sequencing technologies, allow one to infer mRNA isoforms but have limitations for direct understanding of the complexity of underlying transcript isoforms. For example, emerging long read sequencing technologies will be needed to obtain evidence of the connectivity of different promoter isoforms and splice isoforms with 3′ isoforms, knowledge that is mostly unavailable with current platforms. In light of growing mechanistic connections between transcription, splicing, and 3′ end processing, it will be critical in the future to have awareness of full-length transcript structures.

### Complex landscapes of 3′ UTR isoform expression in *Drosophila*

Despite the lack of a next-generation sequencing-based annotation of *Drosophila* APA isoforms, the character of the most utilized PAS was already investigated through analysis of EST data [32, 33]. Strikingly, these early analyses were already able to define the PAS strength hierarchy that we identify in our study. Not surprisingly, the AAUAAA hexamer acts as the canonical PAS in *Drosophila* as in all other higher eukaryotes investigated to date, and as predicted at the time of its discovery in 1976 [55]. We confirm the previous observation of a significant use of the AAUAUA hexamer in *Drosophila* and identify this variant upstream of 10% of all pAs. Interestingly, this PAS does not appear to be significantly present upstream of cleavage sites in either *C. elegans* [34] or humans [35]. This PAS appears to be functional in *Drosophila*, as shown in experiments that mutated the proximal AAUAUA of *Dl* to AAGAGA, causing loss of proximal 3′ UTR isoform expression and sole recognition of the distal PAS [56]. The higher use of the AAUAUA PAS to define 3′ ends in *Drosophila*, and perhaps in insects more generally, raises questions about the nature of the increase in recognition of this specific variant. Future studies will be needed to understand the molecular basis of this increase in recognition, which could occur through a difference in the specificity of the cleavage and polyadenylation machinery in *Drosophila* or the binding of RBPs that cooperate with recognition of this PAS, among several possible mechanisms.

One of the most intriguing aspects of the expression of APA isoforms in eukaryotes is the specific accumulation of isoforms in a tissue- and length-specific manner. This was observed early on through EST data analysis [48, 57], which was subsequently confirmed through sequencing of 3′ end isoforms [34, 35] and RNA-seq analysis [6]. We greatly expand the number of genes that express 3′ UTR isoforms in a tissue-specific manner in *Drosophila*. Confirming our previous observations, head samples express longer 3′ UTR isoforms when compared to all tissues we analyzed, and indeed we identify hundreds of additional genes exhibiting this pattern of expression. However, not all transcripts that express longer isoforms in head samples are head specific, as many of these appear to be expressed in an analogous manner in somatic tissues found in carcass and cell lines. We identify a previously unrecognized program of ovary-specific shortening events that are carried into the maternally deposited transcriptome. As an ovary 3′ UTR shortening program was also observed in zebrafish [23], this raises the possibility of a conserved mechanistic strategy and/or developmental utilization of this APA program. As the 3′ UTRs of these maternally deposited transcripts rapidly lengthen with the advent of zygotic transcription, it will be interesting to investigate whether there is a functional role of APA to specifically modulate the maternal transcriptome during this developmental transition [58].

Germline expression of specific 3′ UTR isoforms appears to be very complex and subject to change in evolution. For example, expression of ovary-specific short isoforms occurs in zebrafish, where these isoforms are found to be the shortest amongst all tissues analyzed, including testis, while in *Drosophila* we observe the shortest 3′ UTR expression pattern in testis. On the other hand, the expression of shorter 3′ UTRs in testis appears to be more conserved amongst metazoan organisms [59–61]; however, these are possibly more labile to evolution of the specific termini. We find that the *Drosophila* testis appears to have relaxed rules regarding its ability to recognize suboptimal PAS motifs that are not utilized in other tissues. In mammals, it has been suggested that an open chromatin state may contribute to the proficiency of testis for efficient utilization of early PAS to generate short 3′ UTRs [60]. Overall, the role that these tissue-specific dynamics play in biology is just beginning to emerge and should continue to be an active field of investigation.

### Conservation and evolution of APA in *Drosophila*

Conservation of sites of cleavage and polyadenylation has been investigated in mammalian systems [35], showing conservation of tissue-specific patterns. Here, we provide 3′ end evidence for head, ovary, and testis of three different species of *Drosophila*, at a depth much greater than previously achieved in studies of evolutionary conservation of APA [35], allowing deeper analysis of site-specific conservation. Our analysis begins to mine this rich dataset, demonstrating great conservation of patterns of longer 3′ UTR expression in heads and shortest in testis. Furthermore, we identify many syntenic positions that are conserved for 3′ end formation between species and observe a strong role of the PAS and DSEs in mediating conservation or change in the degree of pA recognition. This is only the beginning of an analysis that promises to uncover important *cis*-regulatory elements that mediate tissue-specific accumulation of 3′ end isoforms.

A recent study of quantitative trait loci that affect the expression of alternative 3′ UTR isoforms uncovered many sites under genetic control, identifying variants that cluster around pAs as well as putative RBP binding motifs that appear to have a role in specific 3′ end isoform expression [19]. This and our study confirm the importance of *cis*-regulatory elements in modulating the expression of specific isoforms, some of which play significant biological function in vivo [19]. Our comprehensive annotation of 3′ UTRs in *Drosophila* sets the base for using the fly as a model for understanding the regulation of tissue-specific APA and the functional ramifications of this complex phenomenon in multiple aspects of biology.

## Conclusions

In this study, we provide a comprehensive genomewide annotation and analysis of 3' end mRNA isoforms in three Drosophila species, including dozens of developmental/tissue/cell line 3'-seq datasets in the flagship model fruitfly D. melanogaster. We identify >100K novel 3' termini across these species, and dramatically expand the number of genes known to undergo APA. Furthermore, we identify hundreds of additional genes that exhibit developmental and/or tissue specific expression of 3' UTR isoforms, confirming expression of longer isoforms in head and shorter ones in testis while also uncovering specific shortening of 3' UTRs in ovary. Evolutionary comparisons demonstrate broad conservation in overall patterns of tissue specific APA, but also reveal both conservation and divergence of specific cis elements that direct pA recognition. Additionally, we show that novel annotated 3' UTRs participate in conserved post-transcriptional regulatory networks for miRNAs and RBPs. These analyses only scratch the surface of what can be done with these extensive datasets, and we hope the broader scientific community will take advantage of this rich resource to advance our understanding of 3' end formation and the biological implications of developmental and tissue specific 3' UTR expression and regulation. 

## Methods

### Cell and tissue sample preparation

Total RNA was obtained using TRIzol according to manufacturer guidelines. Samples for head were isolated from 2–5-day-old *D. melanogaster* (*Canton S*), *D. yakuba*, or *D. virilis* mated female flies by cutting precisely at the head, which mostly excluded the thoracic ventral nerve cord. Ovary and testis samples were also isolated from 2–5-day-old *D. melanogaster* (Canton S), *D. yakuba*, or *D. virilis* flies. For embryo collection, 2–5-day-old flies were raised on apple juice agar plates on yeast paste for the required time windows (*D. melanogaster*, 0–45′, 45–90′, 90′–6 h, 6–12 h, 12–18 h, 18–24 h; *D. yakuba*, 0–12 h, 12–24 h; *D. virilis*, 0–12 h, 12–24 h, 24–36 h, 36–48 h). Whole flies were 2–5-day-old mated males or females. Carcass samples of 2–5-day-old females or males were obtained by cutting off and excluding the head and by removing either the ovaries or the testes. OSS cells were cultured as described [62] and the remainder of cell line RNA samples were obtained as part of the modENCODE project [63]. The same RNA samples were used for preparing RNA-seq and 3′-seq libraries.

### 3′-Seq library preparation, mapping, and atlas generation

We used our recently published strategy to generate 3′-seq libraries and the computational workflow to analyze the data [29]. Briefly, 3′-seq libraries were prepared using 2 μg of total RNA as starting material. The total RNA was chemically fragmented and custom oligo-dT primers were used to capture and synthesize cDNA representing the junction of the poly(A) tail and the 3′ end of RNAs. The cDNAs were sequenced using an Illumina HiSeq-1000 sequencer with SE-50 mode. The data were mapped onto the genome assemblies and 3′ end clusters were derived and quantified within a 25-bp window as described [29].

### RNA-seq library preparation and mapping

Total RNA-seq libraries were prepared using the Illumina TruSeq Stranded Total RNA Library Prep Kit (catalog number RS-122-2201) starting with 1 μg of total RNA in water. The protocol was followed exactly as per the manufacturer’s instructions. The libraries were sequenced on an Illumina HiSeq-1000 sequencer. Total RNA-seq data generated from the current study as well as the available modENCODE RNA-seq data (SRP001696) were mapped to the corresponding UCSC genome assemblies: *Drosophila melanogaster* (dm6), *Drosophila yakuba* (dyak3), and *Drosophila virilis* (dvir3). HISAT2 aligner was used for the alignment with default parameters [64].

### Expression quantification

Gene expression was quantified using RNA-seq or 3′-seq employing the trimmed mean of M-value (TMM) normalization method using the edgeR/Limma Bioconductor library [65]. The voom method of Limma [66] was used to correct for the Poisson noise due to the discrete counts of RNA-seq. Gene counts of either RNA-seq or 3′-seq, using our extended annotation (Additional file 5: Table S3), were computed using FeatureCounts [67]. Differential gene expression analysis was performed using a standard workflow using the DESeq2 [68] package in Bioconductor.

### Assignment of 3′ ends to genomic features

3′ Ends were assigned to genomic features in the order 3′ UTR > CDS > 5′ UTR > intron. For genes that had more than one 3′ UTR annotation, the 3′ UTR with the most distal 5′ start coupled with the furthest 3′ end annotation was classified as the terminal 3′ UTR and was used in the downstream analysis. 3′ UTR annotations that result from recognition of pAs in introns were excluded from the analysis but are reported in Additional file 3: Table S2. If an end overlapped two different gene loci in the same orientation, the end was classified as ambiguous and excluded from downstream analysis. If the end did not overlap any existing annotation it was initially flagged as intergenic. We rescued ends falling 3′ of an annotation within a 5-kb window if a matched RNAseq sample had continuous coverage in the window between the annotation and the 3′ end. The coverage was assessed using isoSCM [16], creating annotations of 3′ UTRs based on RNAseq allowing for gaps that are less than 100 nucleotides. These ends set the 3′ limit of our extended terminal 3′ UTR annotation (Additional file 5: Table S3).

### Analysis of features around 3′ ends

To assess sequence composition around cleavage sites, nucleotide distributions were computed around the cleavage site. To identify the most common PAS upstream or DSE downstream of the cleavage site, we looked for the most represented hexamer in a 50-nucleotide window upstream or downstream of the cleavage site. Once this was identified, we removed those ends and repeated the process again until we identified the top ten enriched hexamers for either PAS or DSE.

To compare structure-forming potentials of distal and proximal PAS, the distances between canonical PAS, AAUAAA, and poly(A) cleavage sites were examined within 30, 50, and 100 nucleotides separately. To avoid influence of the weaker PAS, we required no major top ten PAS were found in shorter distances when examining the longer distances. We predicted structures using RNAfold in the ViennaRNA package [69] and the ensemble free energy was calculated for each sequence with a parameter of –p0 –noPS and normalized by the sequence length. As a control, we shuffled each sequence by preserving the dinucleotide composition.

### Identification of genes that express alternative 3′ UTR isoforms

For this and all downstream analyses, we analyzed 3′ ends in the 3′-most terminal 3′ UTR as described above, and we additionally excluded all genes that contain annotated introns in the 3′ UTR. To assess the pattern of 3′ UTR expression for a given gene, we calculated a weighted 3′ UTR length metric, as previously reported [23]. Briefly, we took the average of all 3′ UTR isoform lengths per gene weighted by the 3′-seq expression contribution of each isoform. The genes expressing alternative 3′ UTR isoforms between two samples were defined by a differential weighted 3′ UTR length greater than 100 bp and an expression of greater than 5 RPM in both samples.

### Analysis of 3′ end relative strength

We calculated relative strength scores for each 3′ end in terminal 3′ UTRs undergoing APA. The relative strength at each site was calculated by normalizing expression of the end by the total expression at the site and all downstream recognized sites. A relative strength of 1 signifies that no isoforms for the specific gene are detected beyond that site (max strength) and a score of 0 means that the site is not recognized while downstream ones are. We calculated the difference in strength between the same pA site in two different samples by taking the difference of the relative strength scores for the ends that were recognized in both samples being compared.

### Analysis of orthologous 3′ ends

For this analysis, we only considered ends that had as evidence at least three reads in the head, ovary, and testis libraries, as these three tissues were sequenced in all three species. To identify the syntenic sites between *D. melanogaster* and *D. yakuba* or *D. virilis* we used the UCSC liftOver tool. pA sites that were ±25 nucleotides from the lifted *D. melanogaster* end and were reciprocal best matches when the proposed syntenic site from the other species was lifted back onto the *D. melanogaster* genome were considered functionally conserved. Additionally, we only analyzed ends on terminal 3′ UTRs that have defined orthologs in all three species (9844 genes; Additional file 22: Table S15).

To identify the genes that show a similar pattern of 3′ UTR expression between tissues, we considered the genes that are orthologous in all three species and that are expressed at 5 RPM minimum in both tissues and in all three species.

### miRNA target and RBP motif prediction

We used TargetScanS software [70] to predict miRNA target sites in terminal 3′ UTRs with extended 3′ ends derived in this study (Additional file 6: Table S4). For the deeply conserved miRNA sites, site conservation is defined by 9 out of these 12 species (*D. melanogaster*, *D. simulans*, *D. sechellia*, *D. yakuba*, *D. erecta*, *D. ananassae*, *D. pseudoobscura*, *D. persimilis*, *D. willistoni*, *D. virilis*, *D. mojavensis*, *D. grimshawi*), at least one of which had to be a *virilis-group* species (*D. virilis*, *D. grimshawi*, *D. mojavensis*). Only the strong seeds 7mer-m8 and 8mer were considered. Position weight matrices (PWMs) for RBPs were taken from [71], and the fimo program in The MEME Suite (http://meme-suite.org/) was used to scan RBP sites in the annotated 3′ UTRs. The RBP site conservation is defined by presence in at least 3/5 *melanogaster-subgroup* species (*D. melanogaster*, *D. simulans*, *D. sechellia*, *D. yakuba*, and *D. erecta*) along with at least one *obscura-group* species (*D. pseudoobscura*, *D. persimilis*) with one mismatch allowed.

### Data accessibility

The datasets generated and analyzed during the current study are available at the NCBI GEO repository (GSE101603) [72].

## Additional files

Additional file 1: Figures S1–S12. **Figure S1.** Correlation of gene expression measurements derived using either RNA-seq or 3′-seq of matched ovary or testis samples. **Figure S2.** Principal component analysis of 3′-seq and RNA-seq data. **Figure S3.** Distribution of reads and 3′ ends onto genomic features. **Figure S4.** Examples of a gene with diverse APA patterns. **Figure S5.** Rare events maintain a signature of bona fide 3′ ends. **Figure S6.** Un-scaled analysis of weighted 3′ UTR length. **Figure S7.** Additional examples of APA dynamics in cell lines. **Figure S8.** APA dynamics during embryogenesis. **Figure S9.** Nucleotide distribution around *D. yakuba* and *D. virilis* 3′ ends. **Figure S10.** Numbers of additional miRNA sites and example of deeply conserved targets. **Figure S11.** Nucleotide distribution around *D. melanogaster* 3′ ends subdivided by class. **Figure S12.** Comparison of structural forming potential of the proximal and distal PAS-polyA regions. (PDF 4462 kb)

Additional file 2: Table S1. 3′-Seq and RNA-seq datasets generated and used in this study with corresponding mapping statistics. (XLS 34 kb)

Additional file 3: Table S2. Coordinates of 3′ ends of *D. melanogaster*, *D. yakuba*, and *D. virilis*. Ends are associated with genes and respective genomic features or with intergenic space. Counts supporting the end in each tissue are reported. The tables can be found as gtf files in Additional files 4, 14, and 15. (XLSX 15309 kb)

Additional file 4: GTF file of coordinates of the *D. melanogaster* 3′ ends identified in this study. The attribute field lists the same information as given in Additional file 3: Table S2, including counts for all libraries. (BZ2 1645 kb)

Additional file 5: Table S3. Coordinates of terminal 3′ UTRs for *D. melanogaster*, *D. yakuba*, and *D. virilis* used in this study. Terminal 3′ UTRs that were extended in this study are reported with the new coordinates and flagged as extended when 3′ end and RNA-seq evidence was present to extend existing gene models as described in the “Methods” section. (XLSX 1802 kb)

Additional file 6: Table S4. Coordinates of major 3′ end isoforms of *D. melanogaster* terminal 3′ UTRs. 3′ Ends in terminal 3′ UTRs were filtered according to expression level. Ends are reported here if they constituted at least 5% of total expression detected on the terminal 3′ UTR of each locus in at least one of the sequenced libraries. The table is also available as a gtf file in Additional file 7. (XLSX 1011 kb)

Additional file 7: GTF file of coordinates of the *D. melanogaster* major terminal 3′ UTR isoform ends as given in Additional file 6: Table S4. (BZ2 243 kb)

Additional file 8: Table S5. List of genes that are longer in *D. melanogaster* head compared to ovary or testis. The genes for which the weighted 3′ UTR length is more than 100 bp in head compared to ovary or testis are reported. The weighted 3′ UTR length in each tissue and the difference for each comparison are reported. (XLSX 115 kb)

Additional file 9: Table S6. List of gonad-specific shortened genes. The table provides the genes for which the weighted terminal 3′ UTR length is more than 100 bp shorter in ovary compared to both S2 cells and male carcass or more than 100 bp shorter in testis compared to both S2 cells and female carcass. (XLSX 57 kb)

Additional file 10: Table S7. List of head-specific extended genes. The table provides the genes for which the weighted terminal 3′ UTR length is more than 100 bp longer in head compared to both ovary and female carcass. (XLS 42 kb)

Additional file 11: Table S8. List of genes with 3′ UTRs extended or shortened during embryonic development. The table provides the genes whose weighted terminal 3′ UTR length differs by at least 100 bp between early embryo (0–45′) and late embryo (18–24 h). (XLSX 128 kb)

Additional file 12: Table S9. List of genes with weighted 3′ UTR length longer in head compared to male carcass. The genes for which the weighted 3′ UTR length is more than 100 bp in head compared to male carcass are reported. The weighted 3′ UTR length in each tissue and the difference for each comparison are reported. (XLSX 61 kb)

Additional file 13: Table S10. List of genes whose 3′ UTRs are extended in head compared to male carcass and undergo extension during embryogenesis. The gene list refers to the 152 genes highlighted in black in Fig. 3m. (XLSX 39 kb)

Additional file 14: GTF file of coordinates of the *D. yakuba* 3′ ends identified in this study. The attribute field lists the same information as given in Additional file 3: Table S2, including counts for all libraries. (BZ2 507 kb)

Additional file 15: GTF file of coordinates of the *D. virilis* 3′ ends identified in this study. The attribute field lists the same information as given in Additional file 3: Table S2, including counts for all libraries. (BZ2 787 kb)

Additional file 16: Table S11. Conservation of 3′ UTR lengthening in head compared to both ovary and testis in *Drosophila melanogaster/yakuba/virilis.* The gene sets shown in Fig. 4i are reported together with the gene symbol and FlyBase ID. (XLSX 70 kb)

Additional file 17: Table S12. Conservation of 3′ UTR lengthening in head compared to both male carcass and testis in *Drosophila melanogaster/yakuba/virilis.* The gene sets shown in Fig. 4j are reported together with the gene symbol and FlyBase ID. (XLSX 61 kb)

Additional file 18: Table S13. Table of conserved miRNA target sites. A summary of conserved TargetScanS miRNA target predictions in both canonical 3′ UTR and newly annotated 3′ UTRs in this study. See the “Methods” section for the criteria of conservation. (XLSX 252 kb)

Additional file 19: GTF file of coordinates of deeply conserved miRNA target sites. The summary stats for this are given in Additional file 18: Table S13. (BZ2 83 kb)

Additional file 20: Table S14. Table of conserved Elav and Pumilio binding sites. A summary of conserved RBP binding sites for Elav and Pumilio in both canonical 3′ UTR and newly annotated 3′ UTRs in this study. See the “Methods” section for the criteria of conservation. (XLSX 93 kb)

Additional file 21: GTF file of coordinates of conserved RNA binding sites for Pumilio and Elav. The summary stats for this are given in Additional file 20: Table S14. (BZ2 46 kb)

Additional file 22: Table S15. Table of genes that are three way orthologs between *D. melanogaster*, *D. yakuba*, and *D. virilis*. (XLSX 368 kb)
