# Supplementary material for: Landscape and evolution of tissue-specific alternative polyadenylation across Drosophila species
Source: Genome Biol. 2017 Nov 30;18:229. doi: 10.1186/s13059-017-1358-0 (PMC5707805; doi:10.1186/s13059-017-1358-0)
Supplement: Supplementary file 1 — Figure S1. Correlation of gene expression measurements derived using either RNA-seq or 3′-seq of matched ovary or testis samples. Figure S2. Principal component analysis of 3′-seq and RNA-seq data. Figure S3. Distribution of reads and 3′ ends onto genomic features. Figure S4. Examples of a gene with diverse APA patterns. Figure S5. Rare events maintain a signature of bona fide 3′ ends. Figure S6. Un-scaled analysis of weighted 3′ UTR length. Figure S7. Additional examples of APA dynamics in cell lines. Figure S8. APA dynamics during embryogenesis. Figure S9. Nucleotide distribution around D. yakuba and D. virilis 3′ ends. Figure S10. Numbers of additional miRNA sites and example of deeply conserved targets. Figure S11. Nucleotide distribution around D. melanogaster 3′ ends subdivided by class. Figure S12. Comparison of structural forming potential of the proximal and distal PAS-polyA regions. (PDF 4462 kb) [file 13059_2017_1358_MOESM1_ESM.pdf]

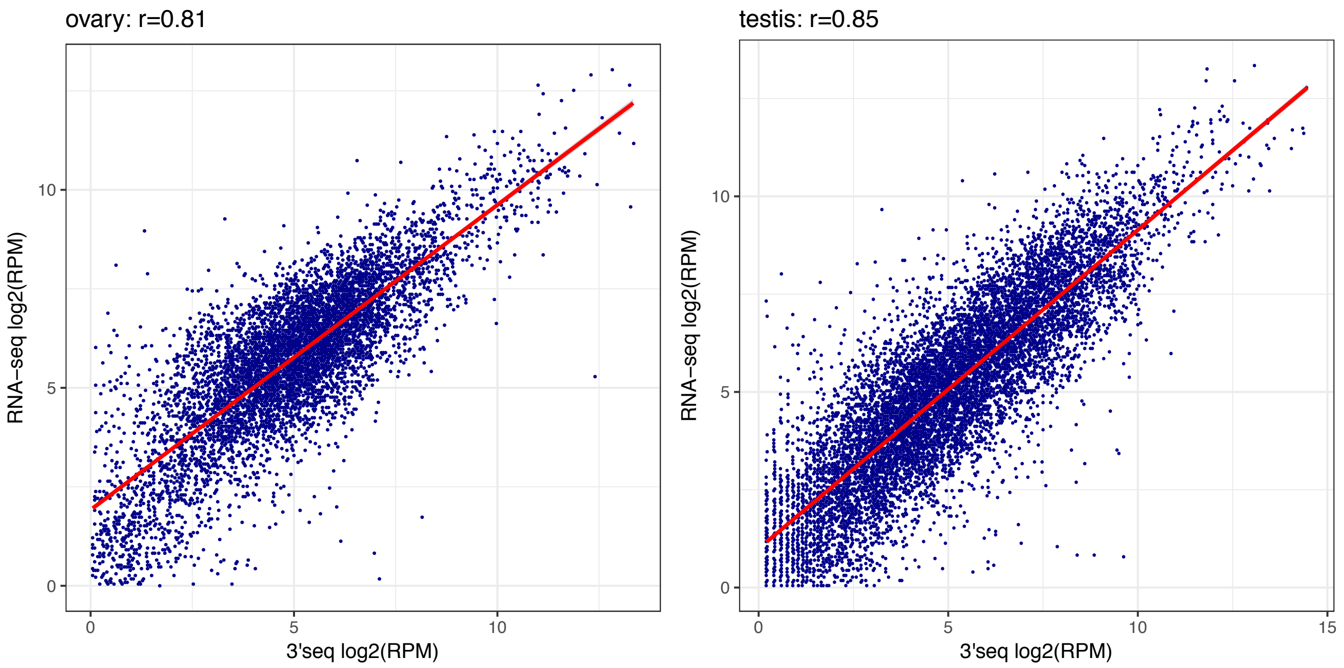

Figure S1. Correlation of gene expression measurements derived either using RNA-seq or 3'-seq of matched ovary or testis samples. Pearson correlation coefficient and p values are shown. Red line represents linear regression fit.

Sanfilippo et al,
Supplementary Figure S1

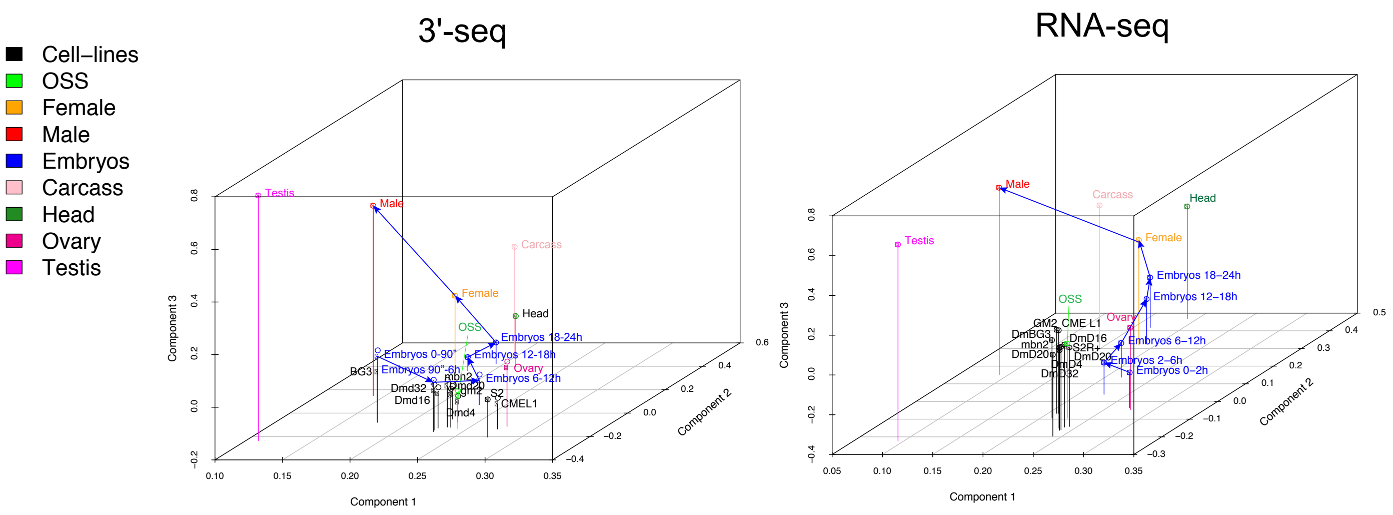

Figure S2. Principal component analysis of 3'-seq and RNA-seq data.
Principal component analysis based on 3'-seq and matching RNA-seq expression form similar clusters, suggesting they have similar expression patterns. All cell lines clearly separate from tissues and an embryonic developmental time course (the arrowed blue lines traces from early embryo to adult body). Ovary is clustered closely with the early embryo 0-2 hr. By contrast, testis, head, and adult body were separated from ovary.

Sanfilippo et al,
Supplementary Figure S2

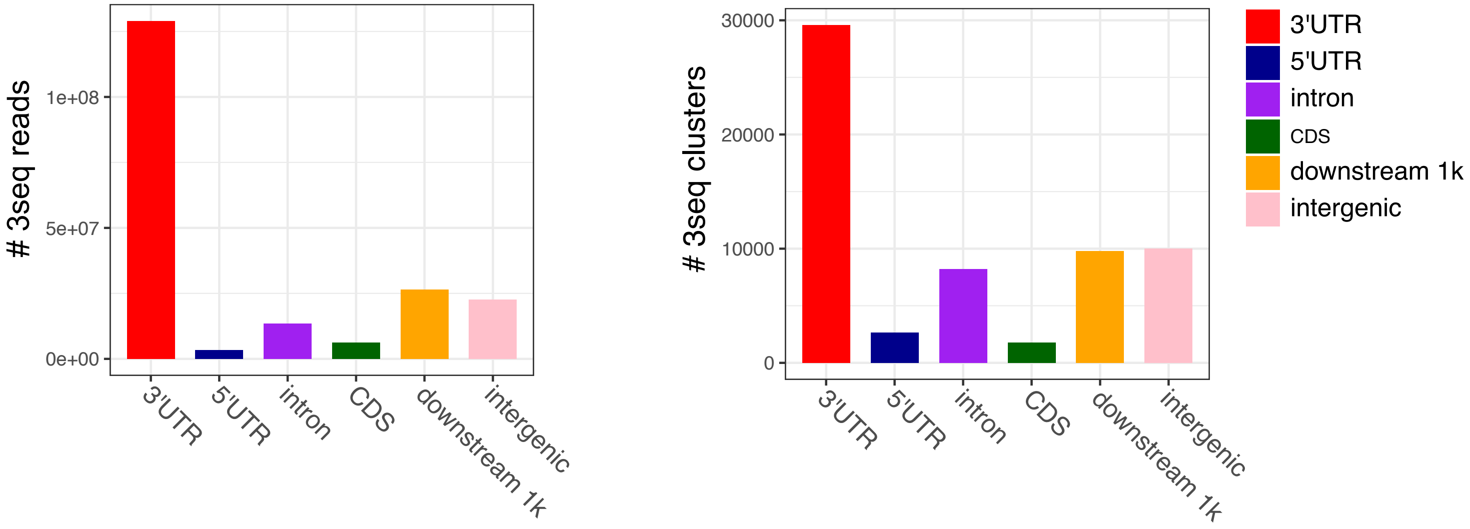

Figure S3. Distribution of reads and 3' ends onto genomic features.
Distribution of raw counts of 3'-seq reads or derived 3' end sites onto genomic features in the *D. melanogaster* annotation (r6.12) and 1kb downstream of annotation.

Sanfilippo et al,
Supplementary Figure S3

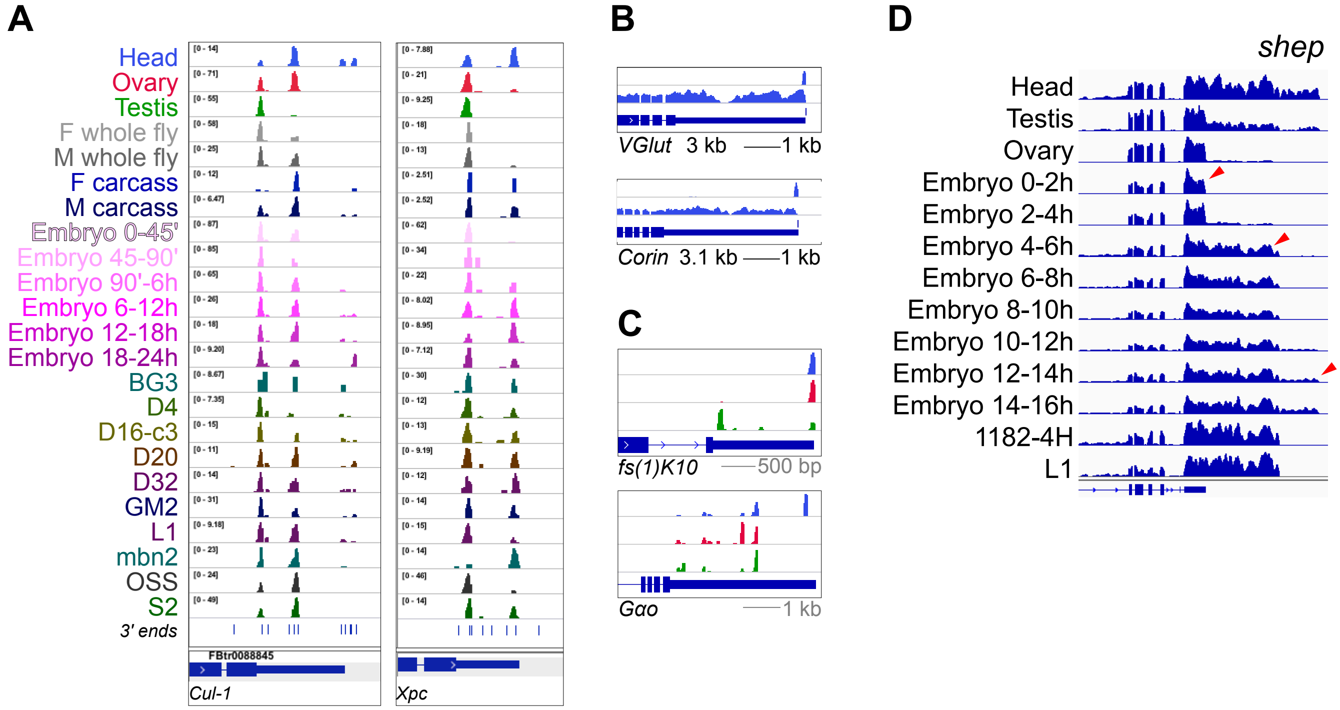

Figure S4. Examples of a gene with diverse APA patterns.
(A) Example of genes with complex pattern of 3' end isoforms expression across adult tissues, embryonic timecourse, and cell lines.
(B) Example of genes with one single long 3' UTR supported by a single 3' end in head.
(C) Example of genes with distinctive tissue-specific APA isoforms deployed in testis, ovary and head.
(D) Example of a gene with distinctive “tiered” expression of progressively longer 3' UTR isoforms during embryogenesis.

Sanfilippo et al,
Supplementary Figure S4

Analysis of 3' end isoform that make up 5% or less of total expression of 3' UTRs

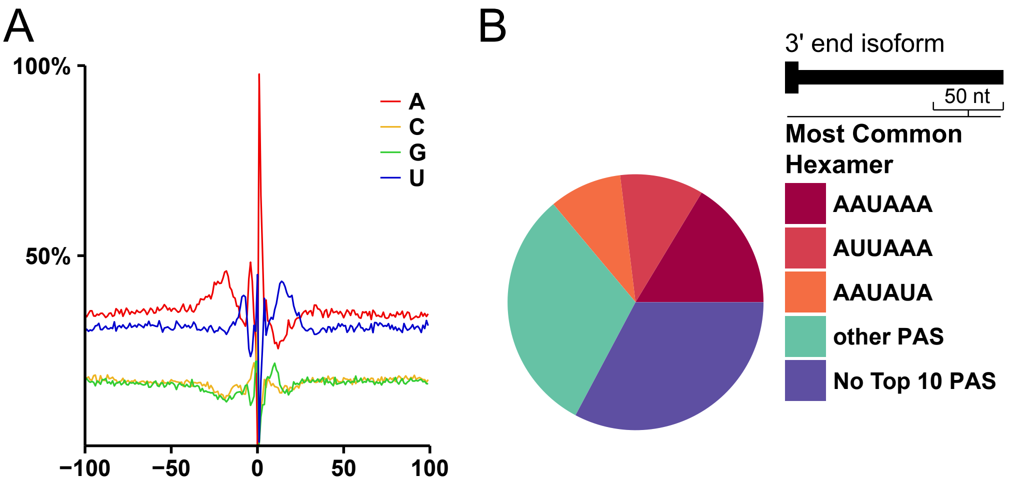

Figure S5. Rare events maintain a signature of bona fide 3' ends.
3' ends of 3' UTR isoforms that were representative of less than 5% of overall expression in the terminal 3' UTRs of each gene were analyzed. (A) 3'-seq derived 3' ends are aligned around the predicted cleavage site and the nucleotide distribution around cleavage site is plotted (centered at 0). % of each of 4 nucleotides in a window of 200 bp centered at the cleavage site are shown.
(B) De-novo derivation of most represented hexamers in a 50 nt window upstream of 3'-seq derived 3' end positions of rare (less than 5% total expression) 3' UTR isoforms.

Sanfilippo et al,
Supplementary Figure S5

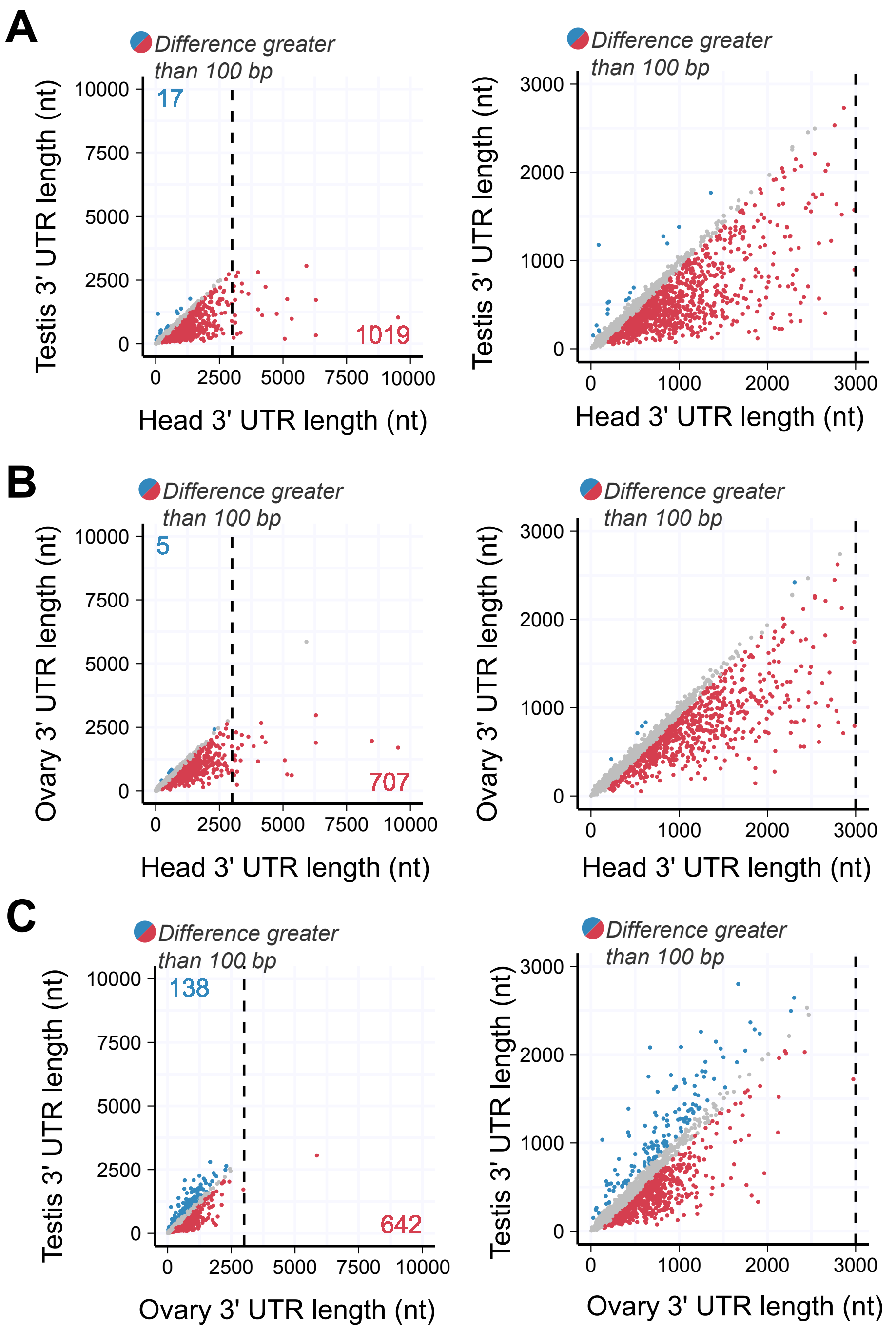

Figure S6. Un-scaled analysis of weighted 3' UTR length
(A-C) Genome-wide patterns of tissue-specific APA revealed by pairwise analyses of weighted 3' UTR length (denoted as 3' UTR length for simplicity) comparison between tissues. Weighted 3' UTR length is obtained taking the average of all 3' UTR isoforms length per gene weighted by the contribution of each isoform expressed. Genes are expressed at a minimum of 5 RPM in all samples. The genes which weighted 3' UTR length differs by 100 bp or more between samples are shown in color, red - longer weighted length in the sample on the x axis, blue - longer weighted length in the sample on the y axis. Shown on the left is the analysis without applying a 3 kb cutoff. Visualizing the data with a 3kb cutoff (right panels) results in the lost of only a few genes (A) Female head vs. testis. (B) Female head vs. ovary. (C) Ovary vs testis.

Sanfilippo et al,
Supplementary Figure S6

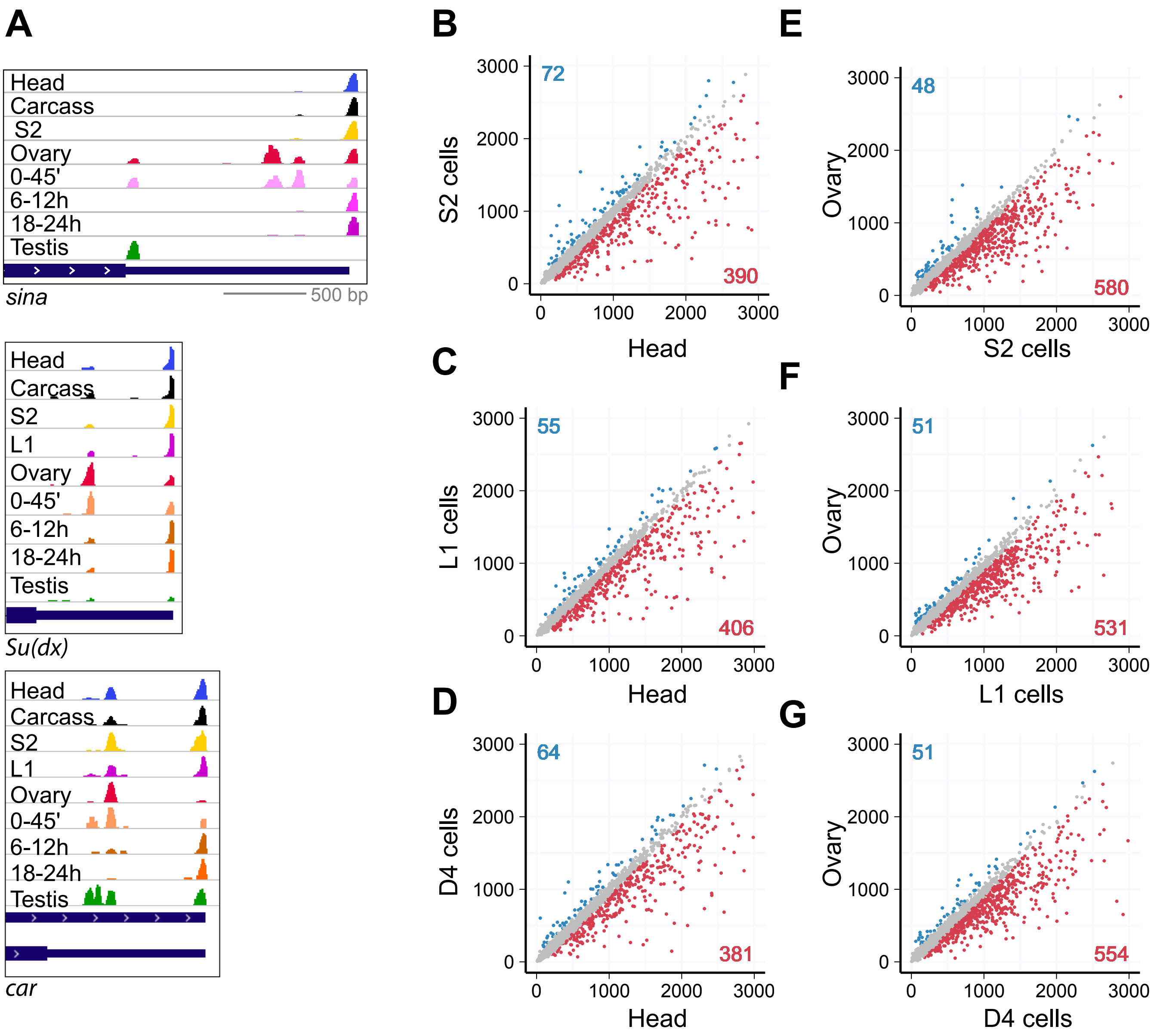

Figure S7. Additional examples of APA dynamics in cell lines and ovary.
(A) Examples of gene that expresses a pattern of 3' UTR isoform expression specific to the ovary and early 0-2h embryo, compared to a range of other developmental stages, tissues, and cell lines.
(B-G) Genome-wide patterns of tissue-specific APA revealed by pairwise analyses of weighted 3' UTR length (denoted as 3' UTR length for simplicity) comparison between tissues. Weighted 3' UTR length is obtained taking the average of all 3' UTR isoforms length per gene weighted by the contribution of each isoform expressed. Genes are expressed at a minimum of 5 RPM in all samples. The genes which weighted 3' UTR length differs by 100 bp or more between samples are shown in color, red - longer weighted length in the sample on the x axis, blue - longer weighted length in the sample on the y axis. (B-D) Comparison of S2, L1 or D4 cell lines to head. (E-G) Comparison of ovary to S2, L1 or D4 cell lines.

Sanfilippo et al,
Supplementary Figure S7

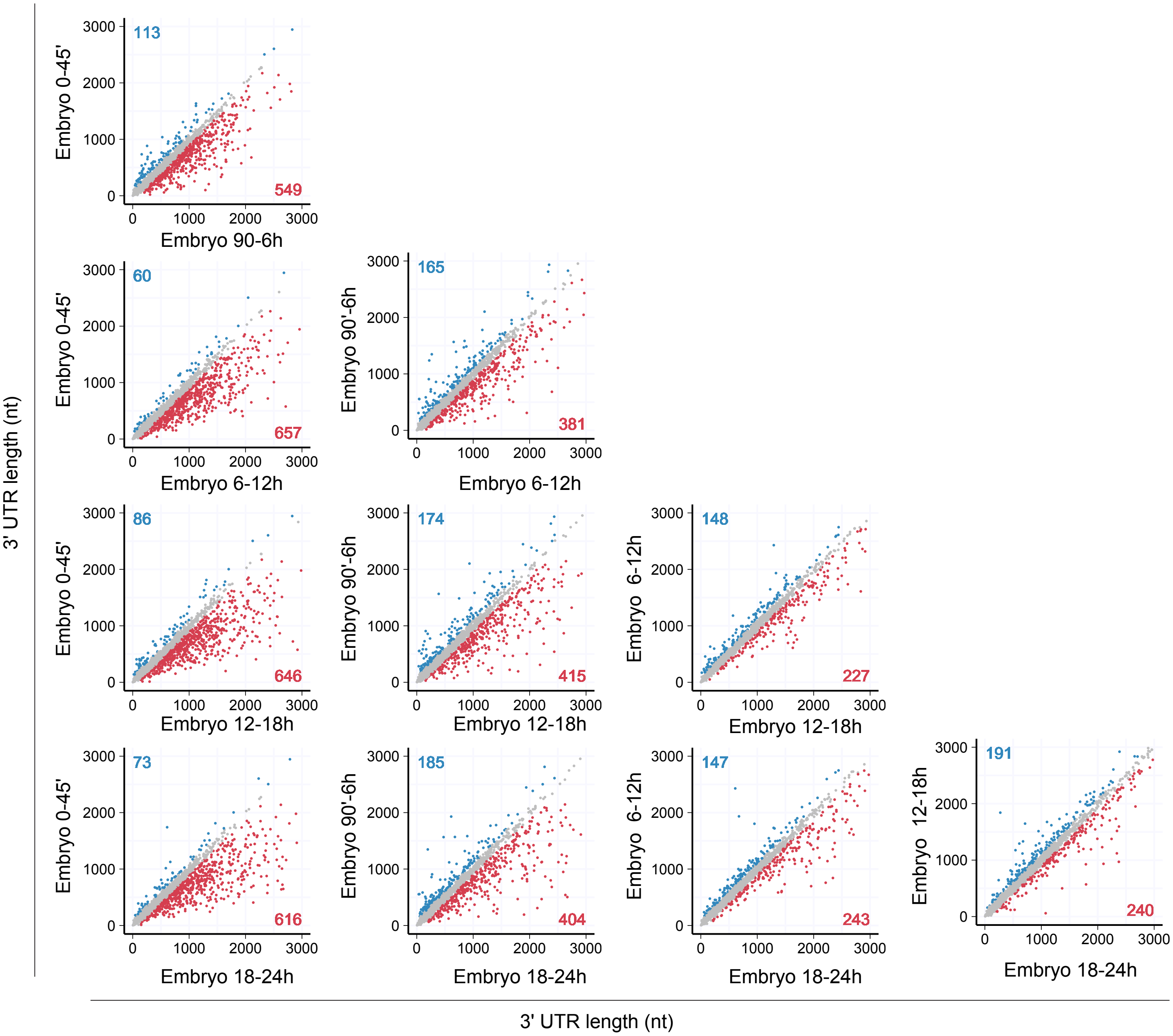

Figure S8. APA dynamics during embryogenesis.
Genome-wide patterns of tissue-specific APA revealed by pairwise analyses of weighted 3' UTR length (denoted as 3' UTR length for simplicity) comparison between tissues. Weighted 3' UTR length is obtained taking the average of all 3' UTR isoforms length per gene weighted by the contribution of each isoform expressed. Genes are expressed at a minimum of 5 RPM in all samples. The genes which weighted 3' UTR length differs by 100 bp or more between samples are shown in color, red - longer weighted length in the sample on the x axis, blue - longer weighted length in the sample on the y axis. Pairwise comparisons of 3'-seq libraries obtained from different stages throughout embryogenesis.

Sanfilippo et al,
Supplementary Figure S8

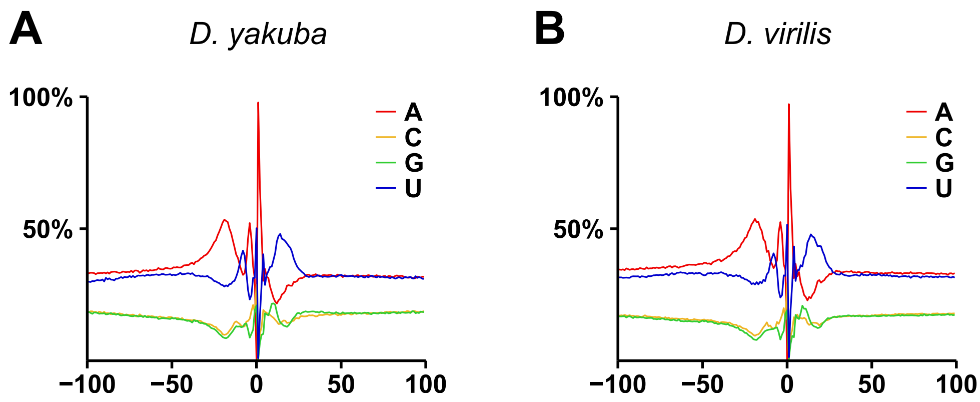

Figure S9. Nucleotide distribution around D. yakuba and D. virilis 3' ends. 3'-seq derived 3' ends are aligned around the predicted cleavage site and the nucleotide distribution around putative cleavage site is plotted (centered at 0). % of each of 4 nucleotides in a window of 200 bp centered at the cleavage site are shown for sites from D. yakuba (A) or D. virilis (B).

Sanfilippo et al,
Supplementary Figure S9

A

conservation criteria	region	#mirs	#genes	#sites	#sites/mir
3/5 in dmel; 1/2 in obscura	flybase.v6 3'UTR	94	5,429	26,647	283
3/5 in dmel; 1/2 in obscura	flybase.v6 3'UTR + extensions	94	5,778	28,259	301
3/5 in dmel; 1/2 in obscura	TargetScan 3'UTR	94	4,378	15,543	165
8 /12 species	flybase.v6 3'UTR	94	4,647	20,529	218
8 /12 species	flybase.v6 3'UTR + extensions	94	4,925	21,601	230
8 /12 species	TargetScan 3'UTR	94	3,753	12,111	129
9 /12; 1 in dmoj/dvir/dgrim	flybase.v6 3'UTR	94	3,497	13,113	140
9 /12; 1 in dmoj/dvir/dgrim	flybase.v6 3'UTR + extensions	94	3,700	13,702	146
9 /12; 1 in dmoj/dvir/dgrim	TargetScan 3'UTR	94	2,756	7,661	82

B

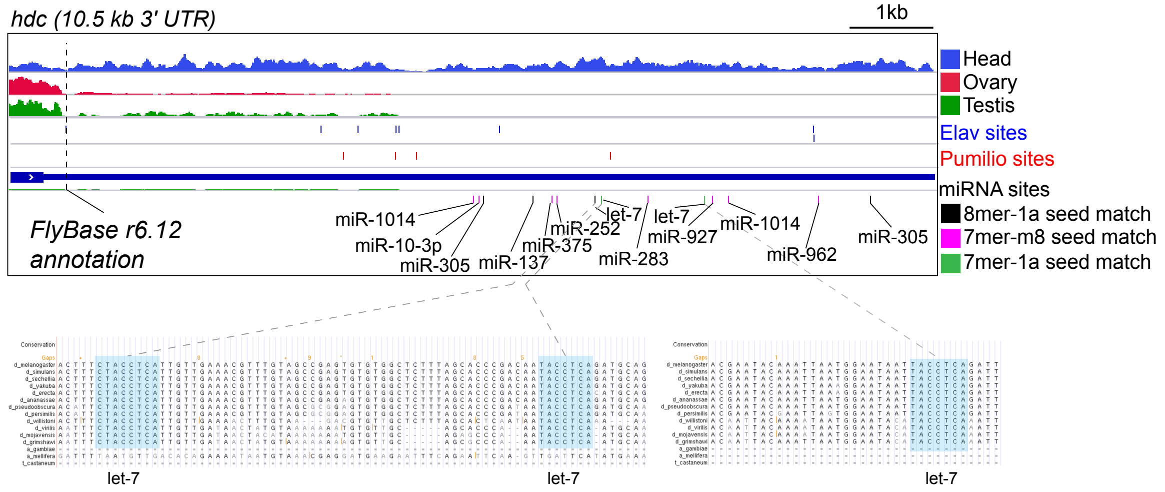

Figure S10. Numbers of additional miRNA sites and example of deeply conserved targets.
(A) We used TargetScanS algorithm to perform target predictions using the 3' UTR databases used in the current available releases of TargetScanFly (v6.2), FlyBase (r6.12) and our current 3' UTR annotations based on 3'-seq atlas (FlyBase+ext). This table supports the graph in Figure 5A, which reports only 7mer-m8 seed matches to pan-Drosophilid conserved miRNAs, bearing conservation properties across the indicated fly species. (B) Example of headcase (hdc), FlyBase (r6.12) annotates no miRNA binding sites in the short 3' UTR. However, an abundance of deeply conserved miRNA, Elav and Pumilio binding sites exists in the much longer 3' UTR that is well-expressed in head. The 3' UTR harbors 3 deeply conserved sites in 12/12 species for let-7 as shown in the insets.

Sanfilippo et al,
Supplementary Figure S10

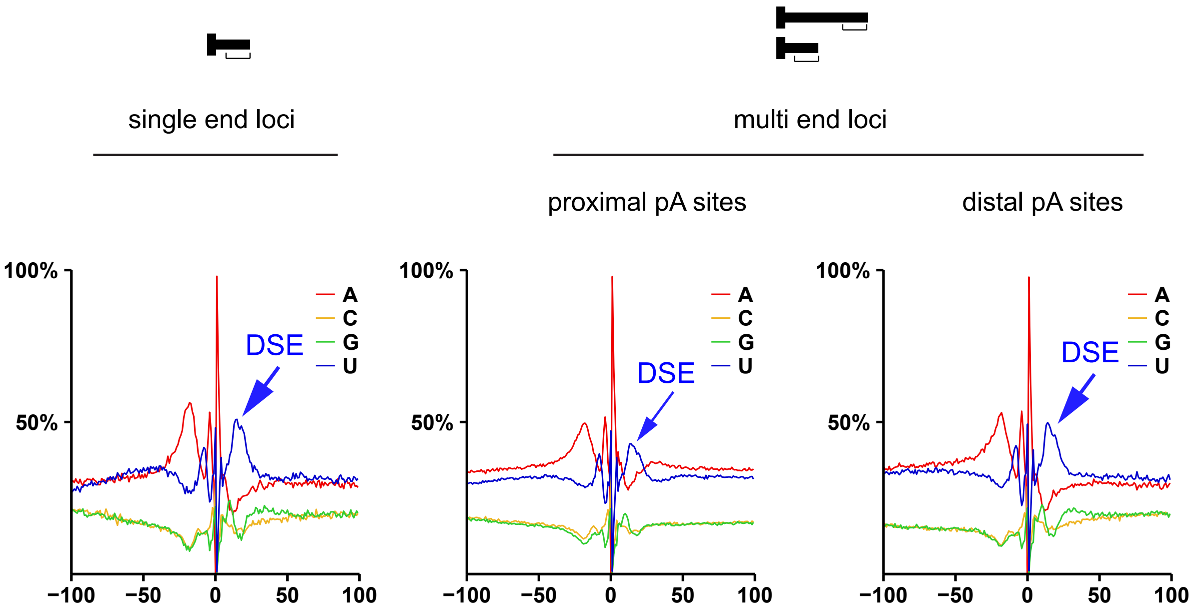

Figure S11. Nucleotide distribution around D. melanogaster 3' ends subdivided by class.
3'-seq derived 3' ends are aligned around the predicted cleavage site and the nucleotide distribution around putative cleavage site is plotted (centered at 0). % of each of 4 nucleotides in a window of 200 bp centered at the cleavage site are shown for sites from D. melanogaster. The sites were divided between ends from single and multi end genes. Multi end gene ends were further subdivided between proximal and terminal ends.

Sanfilippo et al,
Supplementary Figure S11

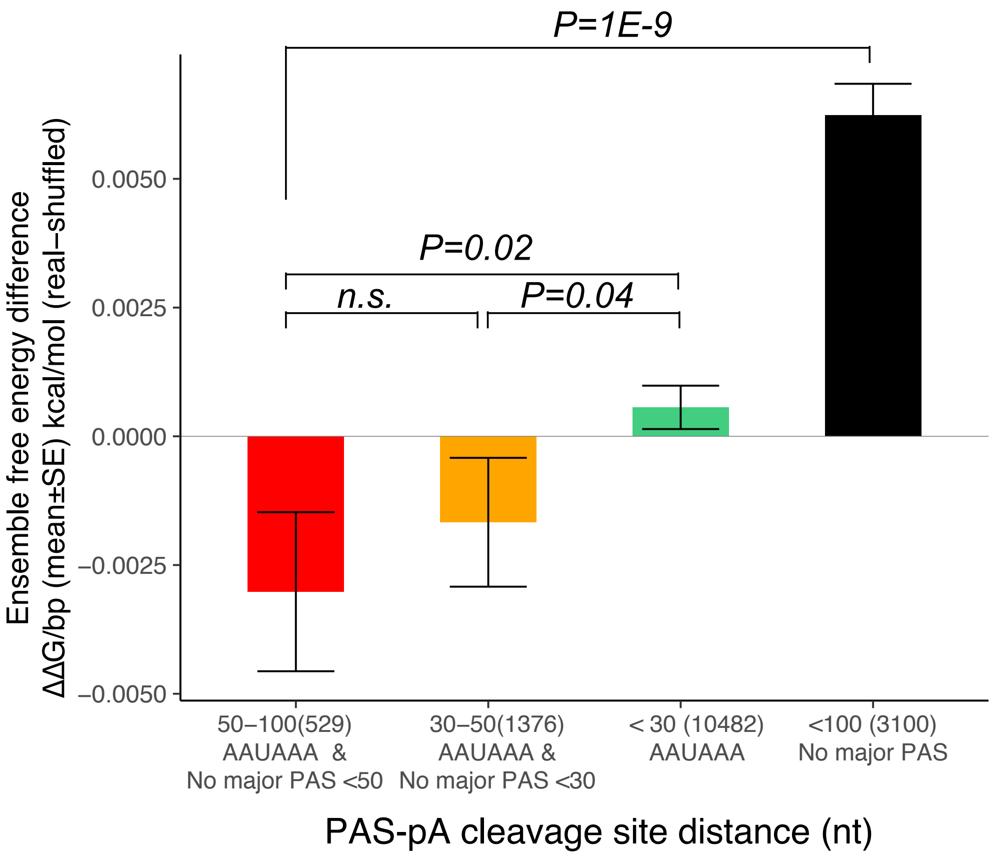

Figure S12. Comparison of structural forming potential of the proximal and distal PAS-polyA regions.
The distances between canonical PAS (AAUAAA), and pA cleavage sites were split into 30nt, 50nt, and 100nt (x-axis). To avoid a bias due to the weaker PAS, we required that no major top 10 PAS be found in the remainder sequence upstream of the pA site. The ensemble free energy (ΔG) were calculated using RNAfold for each PAS-polyA sequence, normalized by sequence length, and then were subtracted from ΔG calculated for dinucleotide shuffled sequences, giving $\Delta\Delta$G/bp as y-axis. On average, PAS at greater distance from pA cleavage site (> 30nt) show more stable structure forming potential than the closer PAS (< 30nt), and no PAS within 100nt show no structure forming potentials compared to the random sequences. Wilcoxon rank-sum test was used to test statistical significance.

Sanfilippo et al,
Supplementary Figure S12
